# Production of the siderophore lysochelin in rich media through maltose-promoted high-density growth of *Lysobacter* sp. 3655

**DOI:** 10.3389/fmicb.2024.1433983

**Published:** 2024-06-26

**Authors:** Fang Zhang, Jia Liu, Lin Jiang, Yongbiao Zheng, Lingjun Yu, Liangcheng Du

**Affiliations:** ^1^School of Life Sciences, Fujian Normal University, Fuzhou, Fujian, China; ^2^Department of Chemistry, University of Nebraska-Lincoln, Lincoln, NE, United States

**Keywords:** *Lysobacter*, siderophore, lysochelin, regulation, biosynthesis

## Abstract

Siderophores are produced by bacteria in iron-restricted conditions. However, we found maltose could induce the biosynthesis of the siderophore lysochelin in *Lysobacter* sp. 3655 in rich media that are not compatible with siderophore production. Maltose markedly promoted cell growth, with over 300% increase in cell density (OD_600_) when LB medium was added with maltose (LBM). While lysochelin was not detectable when OD_600_ in LBM was below 5.0, the siderophore was clearly produced when OD_600_ reached 7.5 and dramatically increased when OD_600_ was 15.0. Coincidently, the transcription of lysochelin biosynthesis genes was remarkably enhanced following the increase of OD_600_. Conversely, the iron concentration in the cell culture dropped to 1.2 μM when OD_600_ reached 15.0, which was 6-fold lower than that in the starting medium. Moreover, mutants of the maltose-utilizing genes (*orf2677* and *orf2678*) or quorum-sensing related gene *orf644* significantly lowered the lysochelin yield. Transcriptomics analysis showed that the iron-utilizing/up-taking genes were up-regulated under high cell density. Accordingly, the transcription of lysochelin biosynthetic genes and the yield of lysochelin were stimulated when the iron-utilizing/up-taking genes were deleted. Finally, lysochelin biosynthesis was positively regulated by a TetR regulator (ORF3043). The lysochelin yield in *orf3043* mutant decreased to 50% of that in the wild type and then restored in the complementary strain. Together, this study revealed a previously unrecognized mechanism for lysochelin biosynthetic regulation, by which the siderophore could still be massively produced in *Lysobacter* even grown in a rich culture medium. This finding could find new applications in large-scale production of siderophores in bacteria.

## Introduction

Iron is an essential nutrient for the growth of microorganisms, since it is needed for several fundamental cellular processes and important proteins containing heme group or iron-sulfur cluster (Frausto da Silva and Williams, 2001; Miethke and Marahiel, 2007). However, microorganisms could not easily utilize iron due to its extremely low solubility, 10^−18^ M at neutral pH in natural habitat (Payne, 1988). To overcome iron limitation, microbes have evolved to produce siderophores that bind iron with high affinity from surroundings and then migrate iron to inside of cells through specific receptor and transport system (Wandersman and Delepelaire, 2004). Bacteria and fungi, as well as some plants, secret various types of siderophores under iron-deficiency environment (Timofeeva et al., 2022). More than 500 siderophores have been found and 300 of them are structurally characterized (Hider and Kong, 2010). Depending on their chemical nature, siderophores are classified into four general types, catecholate, hydroxamate, carboxylate, and mixed types (Barry and Challis, 2009; Ustiatik et al., 2021; Gao and Bian, 2022).

Due to the high affinity for iron, siderophores exhibit great potential for applications in agriculture, pharmaceutics, and environmental protection. The siderophore-antibiotic conjugation is an attractive strategy to enhance uptake and antibacterial potency against multi-drug resistant pathogens (Caradec et al., 2023). Siderophore-producing microbes have the potential to be developed into eco-friendly and sustainable agents to mitigate plant stresses in degraded lands (Singh et al., 2022). For practical applications, siderophores need to be generated easily with a high yield. Chemical synthesis is an option but usually unfeasible due to the cost of multi-step reactions and low yield of siderophores (Cezard et al., 2015). Therefore, using microbial fermentation for siderophore production is a promising alternative approach. Siderophores are produced only under iron-restricted conditions, which means their biosynthesis is tightly regulated. The culture media are vital for siderophores to be produced on a large scale. Usually, synthetic media are selected to cultivate the microbes for siderophores production due to the ease of controlling the iron concentration in the media (Chincholkar et al., 2007). Several synthetic media have been reported for the production of siderophores, such as minimal medium 1 (MM1) for *Bacillus megaterium* (Ferreira et al., 2019), minimal medium 2 (MM2) for *Streptomyces olivaceus* (Meiwes et al., 1990), minimal medium 9 (MM9) with casamino acids for marine bacteria (Hao et al., 2012), minimal succinate-containing medium (MMS) for *Pseudomonas aeruginosa* (Sasirekha and Srividya, 2016), and Burk's medium for *Azotobacter vinelandii* (Baars et al., 2016). Iron concentration is the key factor for siderophores biosynthesis, and the concentration that inhibits the generation of siderophores depends on the microbe and culture medium. In *Bacillus* sp. PZ-1, the inhibition of siderophore production was observed with the iron concentration above 5 μM, and completely inhibited with the concentration of 100 μM (Yu et al., 2017), but in *Azotobacter vinelandii*, the concentration of initial inhibition and complete inhibition was 1 and 10 μM, respectively (Cornish and Page, 1995). As iron is essential for microbial growth, a low iron concentration decreases cell growth and thus impairs the yield of siderophores. Therefore, it is important to find a balanced iron concentration that is low enough to stimulate siderophore production and, at the same time, not to limit the cell growth to reach a proper biomass, which directly impacts the total yield of siderophores. For *Pseudomonas aeruginosa*, the iron concentration of 1 μM in the culture medium supplemented with suitable carbon source could ensure the cell growth and siderophores production (Sexton and Schuster, 2017). Various carbon sources affect the production of siderophores, for example, succinate causes huge production of pyoverdines from *Pseudomonas* genus (Meyer and Abdallah, 1978; Hoegy et al., 2014; Vindeirinho et al., 2021), and glycerol is the optimal carbon source for the siderophores production in *Bacillus megaterium, Bacillus* sp. PZ-1, and *Escherichia coli* (Valdebenito et al., 2006; Santos et al., 2014; Yu et al., 2017).

In many bacteria, the ferric uptake regulator (Fur) is the main regulator for the biosynthesis and transportation of siderophores. When the intracellular iron reaches a certain concentration, Fur binds to ferric iron and then represses the expression of iron uptake genes directly or indirectly; when the iron concentration becomes limiting, Fur is free from ferric iron binding and the repression by Fur is abolished, allowing for iron uptake into cells (Seo et al., 2014; Beauchene et al., 2015). Other regulators have been identified as well, such as PchR for pyochelin activation and PvdS and FpvI for pyoverdines production in *Pseudomonas aeruginosa* (Schalk et al., 2020).

*Lysobacter* is a genus of rod-shaped, gliding Gram-negative bacteria widely distributed in soil and fresh water (Christensen and Cook, 1978). The genus is recognized as a new source of lead compounds for antibiotics due to their prolific production of various bioactive natural products, including polycyclic tetramate macrolactams, cyclic lipodespsipeptides, cephem-type β-lactams, and phenazines (Yue et al., 2022). *Lysobacter* species also exhibit potential in biocontrol of crop diseases for their potent activity to kill a variety of plant pathogenic bacteria, oomycetes, and fungi (Puopolo et al., 2018). In previous work, our lab reported the siderophore lysochelin isolated from *L. enzymogenes*, which was the first siderophore identified from the *Lysobacter* genus (Miller et al., 2023). Lysochelin is biosynthesized by the *lec* gene cluster through an NRPS (non-ribosomal peptide synthetase)-independent biosynthetic pathway. Its structure contains two units of 2,3-dihydroxybenzoic acid (2,3-DHB) and one unit of spermidine (Miller et al., 2023). Despite the progress, the regulatory mechanism underlying lysochelin production has not been investigated. Here, we found that maltose is a strong inducer of lysochelin production in *Lysobacter* sp. 3655. Maltose promoted *Lysobacter* cell growth to a markedly high density in rich culture media without prior removal of iron. While rich media are not a favorable condition for siderophore production, we found that both the transcription of lysochelin biosynthetic genes and the lysochelin yield were dramatically enhanced when *Lysobacter* cells reached a high density. Further results showed that the maltose-promoted growth significantly reduced the iron concentration in the rich media, which created an iron-restricted environment that stimulated the siderophore biosynthesis in rich media. Moreover, we found that lysochelin production in rich media was related to maltose metabolism and quorum sensing. We also found that a TetR regulator was involved in the regulation of maltose metabolism and lysochelin production. Together, the study revealed a new way for lysochelin biosynthesis in rich media, which could be applied for scale-up production of siderophores.

## Methods and materials

### Bacterial strains, plasmids, and growth conditions

Bacterial strains and plasmids used in this study are shown in Supplementary Table 1. Luria-Bertani (LB) broth medium was used for the growth of *Lysobacter* sp. 3655 and *L. enzymogenes* OH11. LBM (1% tryptone, 0.5% yeast extract, 1% NaCl, and 5% maltose) was used for the growth, siderophore production and RNA extraction of strain 3655 and its mutants. LB supplemented with various carbohydrates [5% sucrose (LBS), 5% glucose (LBG), 1% mannose (LBMA), 1% galactose (LBGA), 1% rhamnose (LBR), and 1% fucose (LBF)] were used to explore the siderophore-producing potential of strain 3655. MM813 (0.4% glucose, 0.3% K_2_HPO_4_, 0.138% NaH_2_PO_4_•H_2_O, 0.1% NH_4_Cl, 0.0144% MgSO_4_, 0.015% KCl, and 0.00111% CaCl_2_) was used for the lysochelin production of *L. enzymogenes* OH11 (Miller et al., 2023). LBM supplemented with various concentrations of FeSO_4_ (final concentration of 0, 1, 5, 10, 20, and 40 μM) was used to evaluate the effect of iron on lysochelin production in strain 3655. LB with different amounts of maltose (0, 0.5, 1.0, 2.5, and 5%, w/v) was also used to assess the effect of maltose on the yield of lysochelin in strain 3655. *Escherichia coli* strain DH 5α was cultured at 37°C in LB medium supplemented with gentamicin (Gm, 50 μg/ml) to propagate plasmids. *E. coli* strain S17 was used for intergeneric conjugation.

### DNA manipulation and *Lysobacter* transformation

Chromosomal DNA and plasmids were isolated from *Lysobacter* sp. 3655 or *E. coli* according to the standard techniques (Sambrook et al., 1989). Database searching and sequence analysis were performed using the online program PSI-BLAST (Altschul et al., 1997). For *Lysobacter* transformation, plasmids were first introduced into the *E. coli* S17 and then transferred to *Lysobacter* sp. 3655 by intergeneric conjugation. The transformants were spread on LB plates with kanamycin (Km, 100 μg/ml) and Gentamicin (Gm, 150 μg/ml). After growing at 30°C for 72 h, the single-crossover colonies were selected and plated on LB plates containing 10% (w/v) sucrose and Km (100 μg/ml), 30°C for 72 h. Then the colonies were transferred to LB plates supplemented with Km (100 μg/ml) or Km (100 μg/ml) + Gm (150 μg/ml). The Km resistant and Gm sensitive colonies were the putative double-crossover mutants, which were selected for PCR verification.

### Primers and PCR

All primers used in this study are listed in Supplementary Table 2. PCRs were carried out using Phanta^®^ Max Super-Fidelity DNA polymerase (Vazyme) or r*Taq* DNA polymerase (Takara). For Phanta DNA polymerase, an initial denaturation at 95°C for 3 min was followed by 30 cycles of amplification (95°C for 15 s, 60°C for 15 s, and 72°C for 1 min), and additional 5 min at 72°C. For r*Taq* DNA polymerase, an initial denaturation at 95°C for 5 min was followed by 30 cycles of amplification (95°C for 30 s, 60°C for 30 s, and 72°C for 1 min), and additional 10 min at 72°C. Considering different DNA templates and primers, the annealing temperature and the elongation time were changed in some cases.

### Construction of deletion mutants through homologous recombination

To construct the deletion mutant of siderophore biosynthetic genes (*orf2903, orf2904, orf2905*, and *orf2906*), maltose catabolic genes (*orf2677, orf2678*, and *orf2742*), and other relevant genes (*orf644, orf4279, orf4990*-*orf4993*, and *orf3043*), the DNA fragments corresponding to the upstream and downstream region of above genes were amplified by using respective primers. Then the upstream region was treated either with *Xho*I and *Hin*dIII (for *orf2903, orf2904, orf2905, orf2906, orf644, orf4279, orf4990, orf4992*, and *orf4993*) or with *Apa*I and *Hin*dIII (for *orf2677, orf2678, orf2742, orf3043*, and *orf4991*), and the downstream region was treated with *Hin*dIII and *Spe*I. Each pairs of the digested upstream and downstream DNA fragments were ligated into the *Xho*I/*Spe*I sites or *Apa*I/*Spe*I sites of plasmid pJQ200SK to generate the recombination plasmids. The plasmids were introduced into strain 3655 by intergeneric conjugation. After antibiotic and sucrose screening, the transformants were used for PCR verification by corresponding primers.

For mutant complementation, the DNA fragment containing the upstream region, downstream region and the deletion part of *orf3043* was amplified using primers ORF3043UF/DR. Then the fragment was treated with *Apa*I/*Spe*I and ligated into the same sites of plasmid pJQ200SK to generate the recombination plasmid pJQ200SK::ORF3043C. The recombination plasmid was transferred into ΔORF3043 strain by intergeneric conjugation. After antibiotic and sucrose screening, the transformants were used for PCR verification by primers ORF3043VFO/VRO.

### Growth assay of strain 3655 and its mutants

Strain 3655 and its mutants were cultured in 3 ml of LB for 24 h, then transferred to 25 ml LBM medium, 30°C for 72 h. For the growth assay of strain 3655 in different culture volumes, 1% of the 3 ml culture was transferred to 500 ml flasks containing 25, 50, 100, or 200 ml LBM medium, respectively. The OD_600_ values were determined every 12 h using a spectrophotometer (UV-8000, METASH).

### Lysochelin extraction, HPLC and LC-MS analysis, and quantification

Strain 3655 and its mutants were grown in 3 ml of LB for 24 h, then inoculated to 25 ml LBM medium. The cultures were incubated at 30°C for 72 h. To extract lysochelin, the cultures were extracted with an equal volume of ethyl acetate (containing 0.1% TFA). The ethyl acetate extract was dried using a rotavapor (Buchi, Rotavapor R-200) to afford the crude extract, which was dissolved in 1 ml methanol. A 20 μl aliquot of each of the extracts was analyzed by HPLC (UltiMate 3000, Thermo) using a reversed phase C18 column (Acclaim^TM^ 120 C18, 250 × 4.6 mm), equipped with a UV detector set at 318 nm, with a flow rate of 1 ml/min. The mobile phase comprised solvents A (water containing 0.05% formic acid) and B (acetonitrile containing 0.05% formic acid) using the following gradient elution program: 5–25% B in A for 0–5 min, increased to 80% B at 20 min and maintained for 1 min, then increased to 100% B for 26–28 min, and returned to 5% B in the final 2 min. Lysochelin was confirmed using LC-MS (Thermo, LCQ FLEET). The quantification of lysochelin was based on the HPLC peak aera divided by the OD_600_ value of the same sample. Unless otherwise specified, the yield of lysochelin was presented as fold change in comparison with the yield of the relative control strain such as the wild-type strain cultured in LBM medium.

### RNA isolation and qRT-PCR

RNA was isolated from the wild-type strain 3655 and its mutants cultured in LBM medium for 24 h using SPARKeasy Improved Bacteria RNA Kit (SparkJade). RNA was reverse transcribed to complementary DNA by using EasyScript one-step gDNA removal and cDNA synthesis Supermix (Transgen). qRT-PCR was carried out in LightCycler^®^ 96 Instrument (Roche, Inc) using PerfectStart Green qPCR Supermix (Transgen) with primers listed in Supplementary Table 2. The conditions are used as follows: 94°C for 30 s, followed by 40 cycles of 94°C for 5 s, 60°C for 15 s, and 72°C for 10 s. The 16S rRNA was used as an internal control. The relative transcriptional levels of the genes of interest were normalized to 16S rRNA and determined by using the 2^−Δ*ΔCT*^ method (Livak and Schmittgen, 2001). The values were presented as fold change in comparison with the relative expression levels for each gene at the first test time point in the wild-type strain. Data are presented as the averages of three independent experiments conducted in triplicate.

### Determination of iron concentration

Strain 3655 was cultured in LBM medium at 30°C to the OD_600_ value of 5.0 and 15.0, respectively. The supernatants of two samples were saved by centrifugation (10,000 rpm, 1 min). Then the iron concentrations in LBM medium (used as control) and supernatants were determined using NexION 300D ICP-MS (Perkin Elmer, USA).

### RNA sequencing and transcriptomic analysis

Strain 3655 was grown in LBM medium at 30°C to the OD_600_ value of 5.0 and 15.0, respectively. The cell pellets of two samples were collected by centrifugation (10,000 rpm, 1 min). RNA was extracted via Total RNA Extractor (Trizol) Kit (Sangon, China), and the purify was determined using Qubit 2.0 RNA Detection Kit (Life, USA). To reduce sequencing interference, rRNA was removed by Ribo-off rRNA Depletion Kit (Vazyme, China), and DNA was digested using DNase I (Vazyme, China). The fragmentation, reverse-transcription, 3' ends dA-Tailing, adapter ligation and DNA library construction of purified RNA were performed using VAHTSTM Stranded mRNA-seq Library Prep Kit for Illumina^®^ (Vazyme, China).

The DNA library was sequenced by Illumina NovaSeq 6000, and the raw data were cleaned and evaluated whether the sequencing data was suitable for subsequent analysis. Then the clean reads were spliced through Rockhopper, the CDS prediction and annotation were performed using TransDecoder and NCBI Blast. The expression levels were analyzed using Salmon and WGCNA, and the differentially expressed genes were obtained by DESeq2 with a selection threshold of *q-*value < 0.05 and log2 fold change ≥ 1.0. Genes and Genomes Ontology (GO) and Kyoto Encyclopedia of Genes and Genomes (KEGG) enrichment analysis were performed using topGO and clusterProfiler software, respectively.

### Statistical analysis

All experiments were conducted three times, and all data were expressed as mean ± standard deviation. The SPSS (version 25.0, SPSS Inc., Chicago, ILL, USA) was used for statistical analysis that was performed using one-way analysis of variance (ANOVA) and Tukey's test at *p* < 0.05 or *p* < 0.01.

## Results

### Maltose induces the siderophore lysochelin production in a rich medium

**Figure 1:**
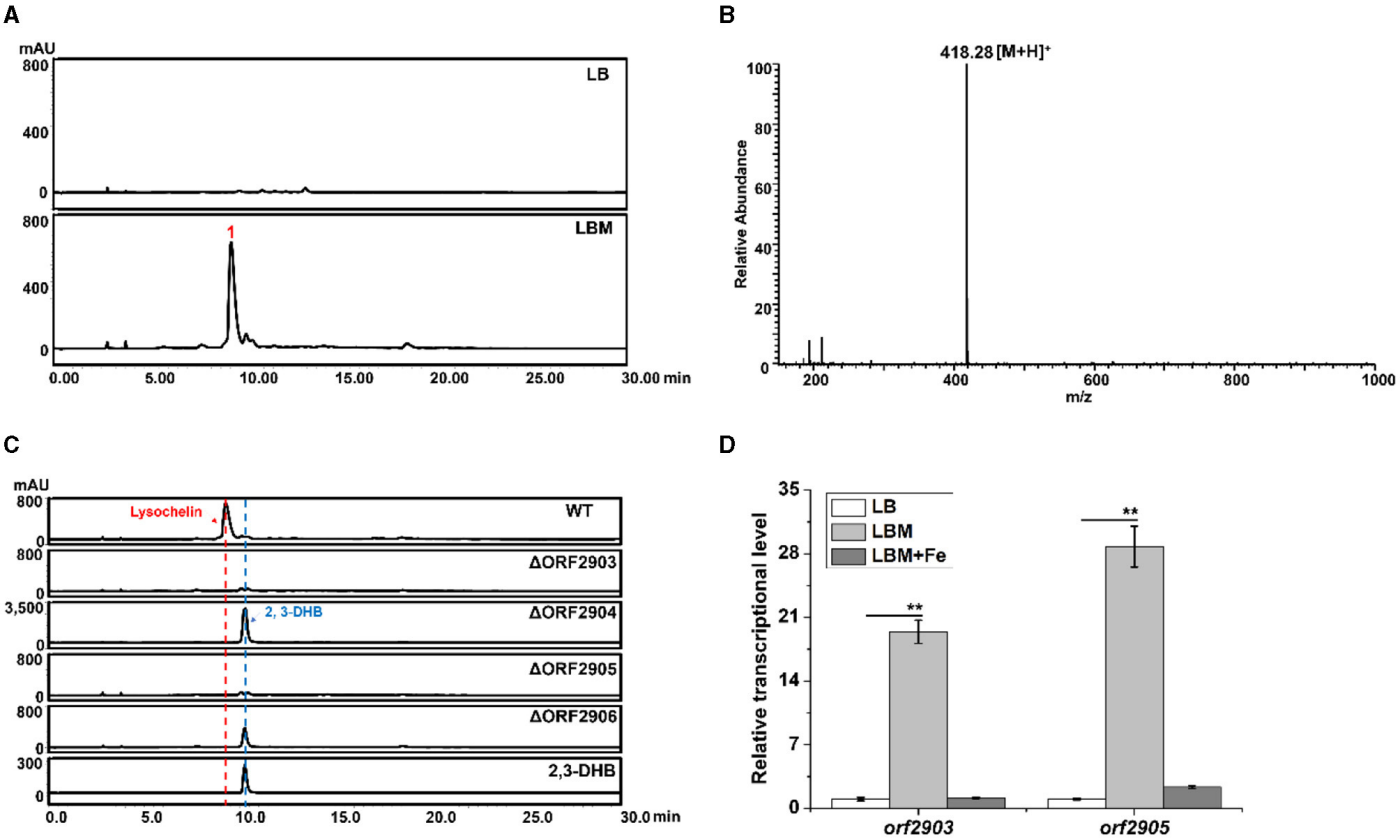
Production of the siderophore lysochelin in rich media. **(A)** HPLC analysis of the culture extracts from *Lysobacter* sp. 3655 wild type (WT) grown in LB medium or LBM medium for 72 h. **(B)** LC-MS analysis of compound **1**. **(C)** HPLC analysis of the culture extracts from WT, deletion mutants ΔORF2903, ΔORF2904, ΔORF2905, and ΔORF2906 grown in LBM medium for 72 h. Standard 2, 3-DHB was included as a reference compound. **(D)** Transcriptional analysis of the lysochelin biosynthetic genes (*orf2903* and *orf2905*) of WT grown in LB medium (white columns), LBM medium (light-gray columns), and LBM with FeSO_4_ (final concentration 20 μM, gray columns) for 24 h, **p < 0.01.

During the study of bioactive natural products in *Lysobacter* sp. 3655, we serendipitously found that adding maltose (5%, w/v) into LB medium (LBM medium) could significantly induce the production of a compound (**1**) with the retention time around 9.3–9.4 min, which was the main product under UV absorption at 318 nm (Figure 1A). This compound was not detectable in the same culture grown in LB medium. Mass spectrometry gave an *m*/*z* 418.28 of [M+H]^+^ (Figure 1B). The retention time and mass of this compound were identical to that of lysochelin, a siderophore recently identified from *L. enzymogenes* OH11 (Supplementary Figure 1; Miller et al., 2023). Lysochelin is biosynthesized through the *lec* gene cluster in *L. enzymogenes* (Miller et al., 2023). We searched for homologous genes of the *lec* cluster in strain 3655 and found Cluster-7, which contains six genes, *orf2903, orf2904, orf2905, orf2906, orf2907*, and *orf2908* that are homologs of *lecA, lecB, lecC, lecD, lecE*, and *lecF*, respectively. The deduced amino acid sequences of the six genes in strain 3655 share an identity of 86, 82, 92, 84, 79, and 84%, respectively, with that of the six *lec* genes in *L. enzymogenes* OH11 (Supplementary Figure 2, Supplementary Table 3). The presence of an entire *lec* homologous cluster in strain 3655 supports that this *Lysobacter* is also a lysochelin producer. To obtain evidence, we generated gene deletion mutants of *orf2903, orf2904, orf2905*, and *orf2906* (Supplementary Figure 3). Indeed, the deletion of these genes abolished the production of compound **1** in LBM medium (Figure 1C), consistent with the compound **1** being lysochelin. Moreover, the 2,3-Dihydroxybenzoate (2,3-DHB), which is the precursor of lysochelin, was massively accumulated in the mutants of *orf2904* and *orf2906* (Figure 1C). The *lecB* homolog *orf2904* encodes (2,3-dihydroxybenzoyl) adenylate synthase, which converts 2,3-DHB to its adenosylation form (2,3-DHB-AMP), and the *lecD* homolog *orf2906* encodes the condensation enzyme that catalyzes two amide bonds between two units of 2,3-DHB-AMP and spermidine, resulting in lysochelin formation (Miller et al., 2023). The results indicated that the biosynthetic pathway of lysochelin in *Lysobacter* sp. 3655 is the same as that in *L. enzymogenes* OH11. Interestingly, the transcriptional level of the lysochelin biosynthetic genes (*orf2903* and *orf2905*) was dramatically increased in LBM medium, whereas the transcription level of the genes in LB medium was barely detectable (Figure 1D). The results show that maltose is a potent inducer of the siderophore lysochelin biosynthesis in *Lysobacter* sp. 3655.

### Maltose promotes vigorous growth of *Lysobacter* sp. 3655 leading to an iron-depleted condition

**Figure 2:**
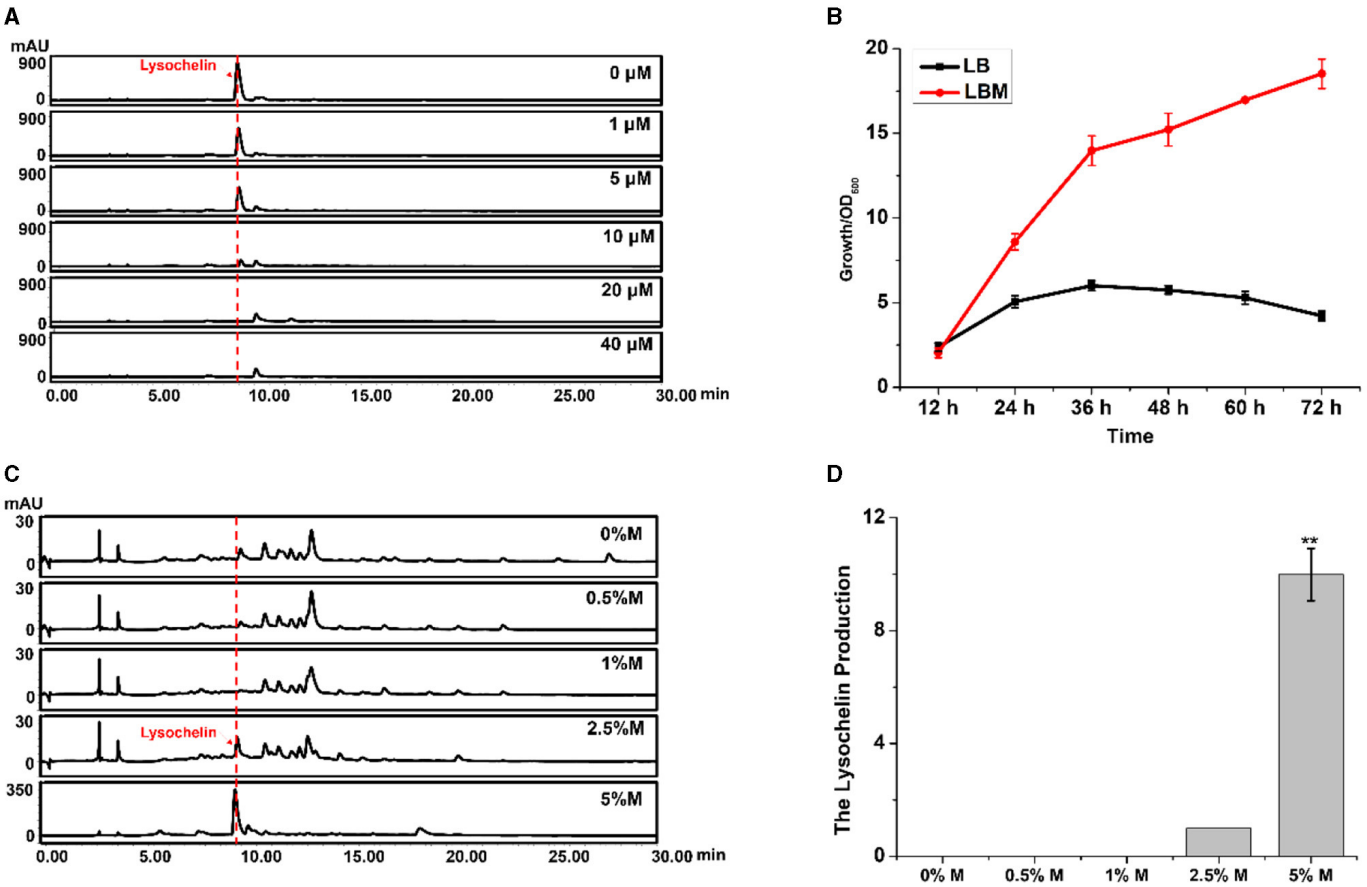
Effects of iron and maltose concentrations on lysochelin production. **(A)** HPLC analysis of lysochelin production in WT grown in LBM medium containing different concentrations of FeSO_4_ (final concentration 0, 1, 5, 10, 20, and 40 μM) for 72 h. **(B)** The growth curve of WT grown in LB medium (black line) and LBM medium (red line). **(C)** HPLC analysis of lysochelin production in WT grown in LB medium containing different concentrations of maltose (0, 0.5, 1, 2.5, and 5%) for 72 h. M, maltose. **(D)** Quantification of lysochelin in the cultures from **(C)**. **p < 0.01.

Siderophores are commonly produced under an iron-limited environment. For example, lysochelin was produced by *L. enzymogenes* grown in a modified minimal medium (MM813) without iron ion (Miller et al., 2023). LB is a eutrophic complex medium, and strain 3655 could not yield lysochelin in this medium (Figure 1A), indicating that LB is not an iron-deficient medium. However, the addition of maltose into LB could dramatically induce lysochelin production (Figure 1A). We proposed two hypotheses, one being that maltose might inhibit iron utilization in strain 3655 that would trigger the strain to synthesize lysochelin to compensate the iron deficiency in cells; the other being that maltose might cause the iron depletion in LB that would induce lysochelin biosynthesis in strain 3655. To obtain evidence, we added iron ion exogenously into LBM medium and evaluated lysochelin production. The results showed that the lysochelin level quickly decreased with the increase of iron concentration. Lysochelin was not detectable when the iron concentration reached 20 μM (Figure 2A). Furthermore, the transcriptional level of *orf2903* and *orf2905* in LBM returned to the level in LB when 20 μM iron was added to LBM (Figure 1D). This suggested that maltose probably might have caused a depletion of iron in LB medium which resulted in lysochelin biosynthesis in this rich medium. We compared the growth of strain 3655 cultured in LB and LBM media. Surprisingly, strain 3655 was flourishing in LBM medium with the maximum OD_600_ value of 18.5 at 72 h, while in LB medium strain 3655 reached its maximum growth at 36 h with the OD_600_ value of 6.0 (Figure 2B). This showed that maltose could dramatically promote the growth of strain 3655, which might have caused the lysochelin induction.

Thus, we evaluated the effect of maltose concentration on lysochelin production. Strain 3655 could not generate lysochelin when the maltose concentration was below 1.0%, while lysochelin was clearly detected when the media contained a higher maltose concentration such as 2.5 and 5.0% (Figures 2C, D). Also, the cell density of strain 3655 was significantly enhanced at maltose concentration of 2.5 and 5.0% (Supplementary Figure 4). Moreover, strain 3655 produced a variable amount of lysochelin when cultured in different volumes of LBM medium. The yield of lysochelin was significantly decreased with the enlargement of cultural volume; the lysochelin production in 200 ml LBM medium decreased 5-fold when compared to that in 25 ml medium (Supplementary Figures 5A, B). Accordingly, the cell density of strain 3655 dramatically reduced when cultured in larger volumes (Supplementary Figure 5C). Together, the results showed that lysochelin biosynthesis was induced at high cell density and abolished at low cell density.

**Figure 3:**
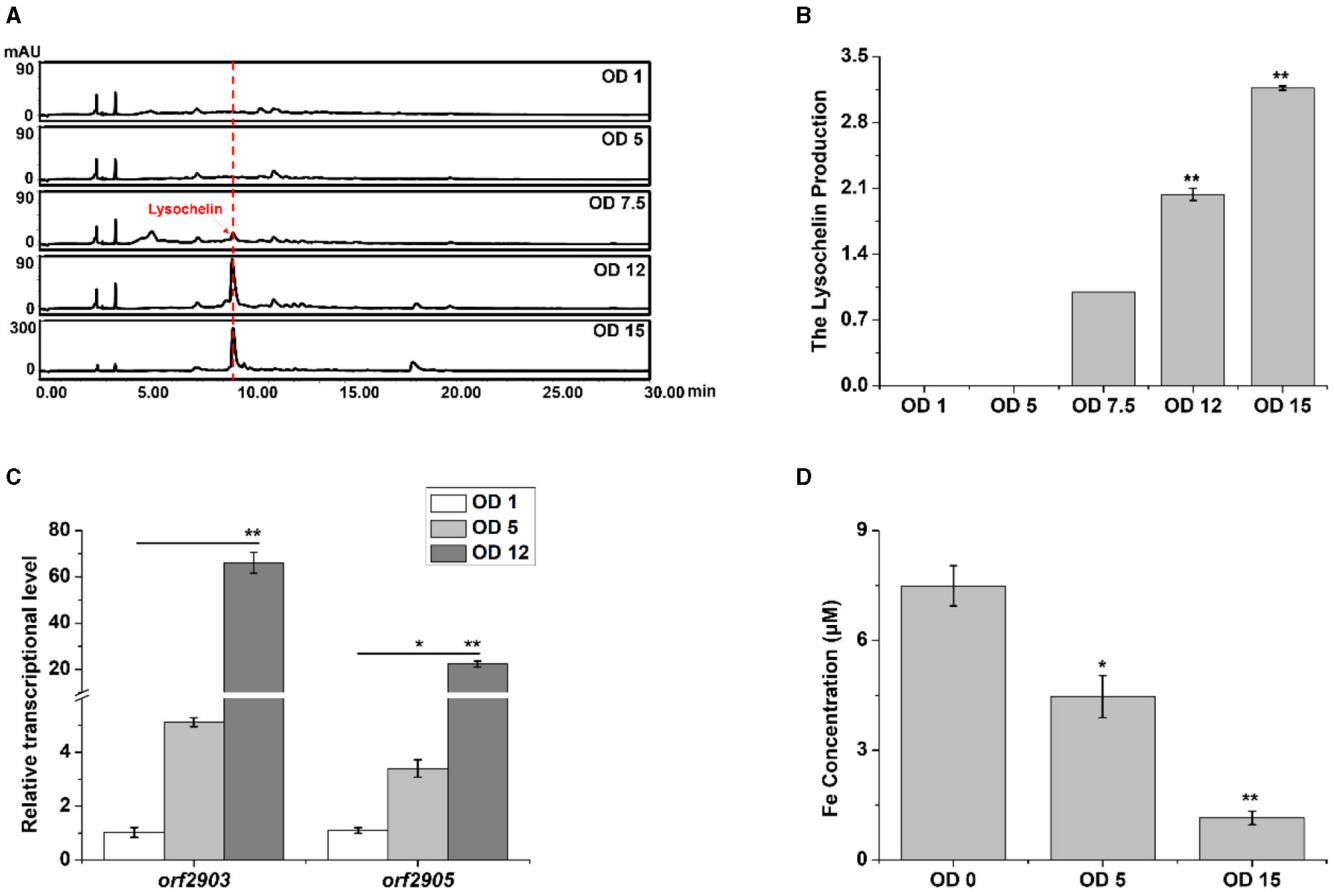
Induction of lysochelin biosynthesis by high cell density. **(A)** HPLC analysis of lysochelin production in WT grown in LBM medium at different OD_600_ values. **(B)** Quantification of lysochelin in the cultures from **(A)**. **(C)** Transcriptional analysis of the lysochelin biosynthetic genes (*orf2903* and *orf2905*) of WT grown in LBM at OD_600_ 1 (white columns), 5 (light-gray columns), and 12 (gray columns). **(D)** The iron concentration in the supernatant of cell cultures at OD_600_ 0, 5, and 15. The blank LBM medium was used as control (OD_600_ 0). *p < 0.05, **p < 0.01.

We investigated the point of growth at which lysochelin could be detected in strain 3655. There was not a detectable amount of lysochelin when the OD_600_ value was 1.0 or 5.0, a detectable amount when the OD_600_ value reached 7.5, and a significant amount when the OD_600_ value increased to 12 and then 15 (Figures 3A, B). Coincidently, the transcriptional level of *orf2903* and *orf2905* at the OD_600_ value of 12.0 was about 20–70-fold higher than that at the OD_600_ value of 1.0 (Figure 3C). We speculated that there was a sufficient amount of iron for the growth of strain 3655 at low cell density and, as a result, the biosynthesis of lysochelin was not turned on. However, as maltose could markedly promote strain 3655 to high-density growth, iron was used up by the “old” cells and the “new” cells would sense the iron-restriction condition, which resulted in lysochelin biosynthesis. To verify the speculation, we determined the iron concentration in LBM medium and the supernatant of cell cultures at OD_600_ 5.0 and 15.0. The results showed that the iron concentration was 7.5 μM in LBM medium, decreased to 4.5 μM in the supernatant of low cell density (OD_600_ value of 5.0), and further reduced to 1.2 μM in the supernatant of high cell density (OD_600_ value of 15.0; Figure 3D). The data strongly supported that maltose induced lysochelin biosynthesis via promoting vigorous growth of strain 3655 to create iron-deficiency for “new” cells.

Next, we examined the effect of other carbohydrates on induction of lysochelin biosynthesis. Glucose could facilitate the high-density growth of strain 3655 (OD_600_ value of 15.0) to produce lysochelin, while sucrose barely promoted the growth (OD_600_ value of 6.0) or lysochelin production (Supplementary Figures 6A, B). Interestingly, adding other carbohydrates, such as mannose, galactose, rhamnose, and fucose into LB medium inhibited the growth of strain 3655 (Supplementary Figure 6C). The results support the notion that lysochelin biosynthesis in strain 3655 might be triggered by the high cell density.

### Lysochelin biosynthesis relates to maltose utilization and quorum sensing

**Figure 4:**
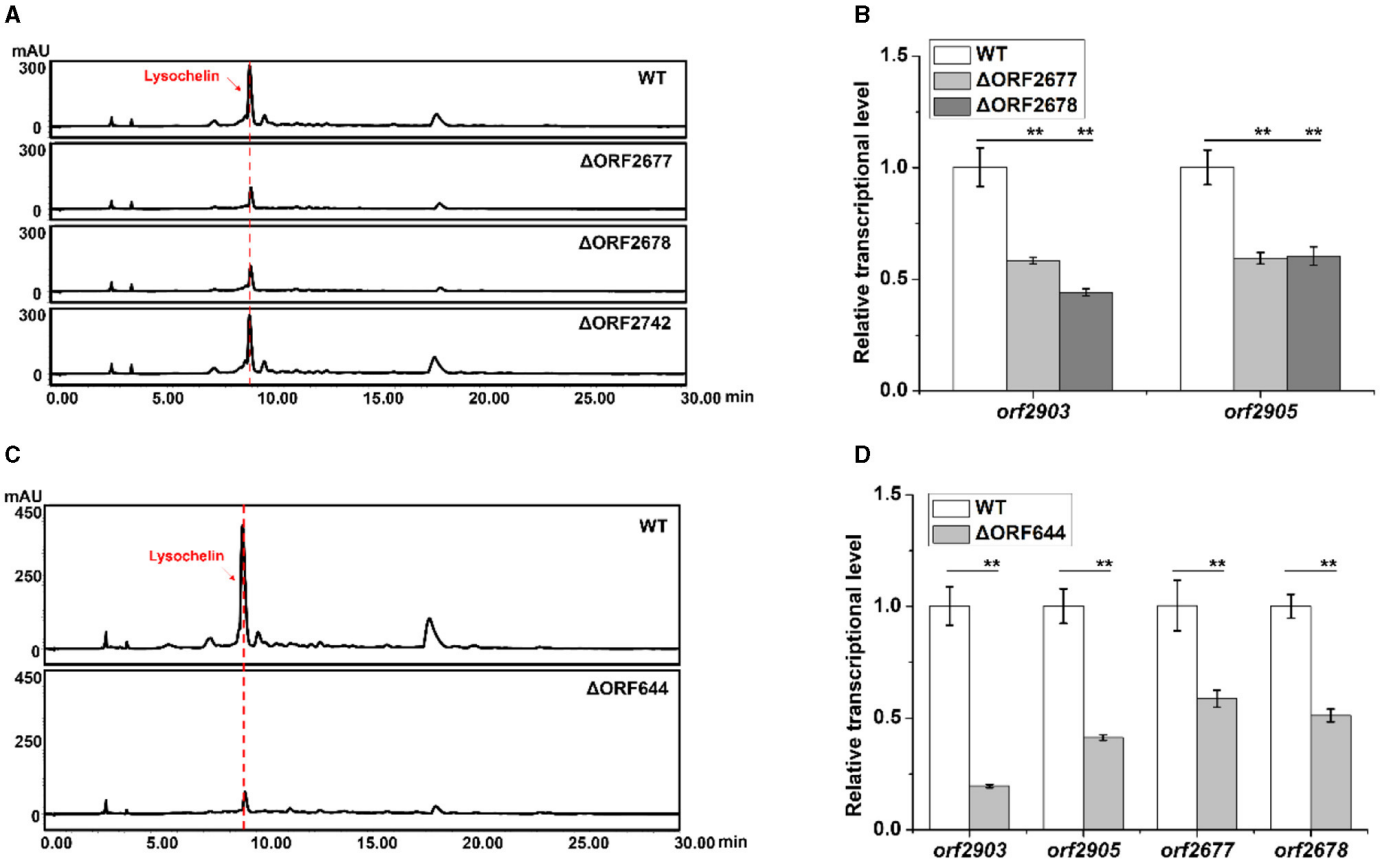
Effects of the maltose metabolism and quorum sensing on lysochelin production. **(A)** HPLC analysis of lysochelin production in WT and the deletion mutants of maltose utilization genes (ΔORF2677, ΔORF2678, and ΔORF2742) grown in LBM medium for 72 h. **(B)** Transcriptional analysis of lysochelin biosynthetic genes (*orf2903* and *orf2905*) of WT (white columns), ΔORF2677 (light-gray columns), and ΔORF2678 (gray columns) grown in LBM for 24 h. **(C)** HPLC analysis of lysochelin production in WT and the deletion mutant of the DF signal biosynthetic gene (ΔORF644) grown in LBM medium for 72 h. **(D)** Transcriptional analysis of *orf2903, orf2905, orf2677*, and *orf2678* of WT (white columns) and ΔORF644 (light-gray columns) grown in LBM for 24 h. **p < 0.01.

Since maltose could induce lysochelin biosynthesis by promoting strain 3655 to high cell density, we investigated the effect of maltose utilization on lysochelin production. Three maltose-utilizing genes *orf2677, orf2678*, and *orf2742* were found through homology search (Supplementary Figures 7–9). The gene *orf2677* encodes a malE-like protein which is a periplasmic maltose binding protein functioning as the primary receptor for the active transport of maltose (Boos and Shuman, 1998). The gene *orf2678* encodes a malF-like protein which is an intrinsic membrane protein of the transport system for maltose (Boos and Shuman, 1998). The gene *orf2742* encodes maltose hydrolase responsible for cleaving maltose into two glucose molecules (Breton et al., 2005). We created the deletion mutants of *orf2677, orf2678*, and *orf2742* (Supplementary Figure 10), and the results showed that the mutants of *orf2677* or *orf2678* significantly reduced lysochelin production, indicating the negative effect of an impaired maltose uptake system on siderophore biosynthesis. The yield of lysochelin in strain ΔORF2677 and ΔORF2678 decreased to 50% of that in the wild-type (WT), and accordingly the cell density of ΔORF2677 and ΔORF2678 was also lowered when compared to that of WT (Figure 4A and Supplementary Figures 11A, B). As expected, the transcriptional level of lysochelin biosynthetic genes *orf2903* and *orf2905* was significantly decreased in strains ΔORF2677 and ΔORF2678 (Figure 4B). However, lysochelin production and cell density of strain ΔORF2742 were similar to that of WT (Figure 4A and Supplementary Figures 11A, B), suggesting that the mutation of this maltose hydrolase alone did not cause a significant impact.

Quorum sensing (QS) is a cell-density-dependent regulatory mechanism mediated by diffusible chemical signal molecules in bacteria (Fuqua et al., 1994). Since lysochelin biosynthesis was connected to cell density, we figured that QS might be involved in the production of lysochelin. Diffusible factors (DF) including 3-HBA (3-hydroxybenzoic acid) and 4-HBA (4-hydroxybenzoic acid) are two QS signals that were previously identified from *L. enzymogenes*, and LenB2, a pteridine-dependent dioxygenase-like protein, is responsible for DF biosynthesis (Qian et al., 2013). In strain 3655, the gene *orf644* encodes a homologous protein of LenB2 with 90% identity in protein sequence (Supplementary Figure 12). To test if the QS signaling is involved in lysochelin production, we constructed a deletion mutant ΔORF644 (Supplementary Figure 13). The cell density and lysochelin production in the mutant decreased to 80% and 22% of that in WT (Figure 4C and Supplementary Figures 11C, D). In accordance with the data, the transcriptional level of *orf2903* and *orf2905* was significantly reduced in mutant ΔORF644 (Figure 4D). Moreover, the deletion of *orf644* decreased the transcriptional level of the maltose uptake genes *orf2677* and *orf2678*, indicating that the DF signaling system might be related to maltose transportation (Figure 4D). Besides, the deletion of *orf644* abolished the production of 3-HBA and 4-HBA, confirming its pivotal role in DF biosynthesis (Supplementary Figure 14). The results showed that the DF signaling system could lower lysochelin production through impairing maltose utilization and high-density growth of strain 3655.

### Transcriptomics analysis identified iron-related genes

**Figure 5:**
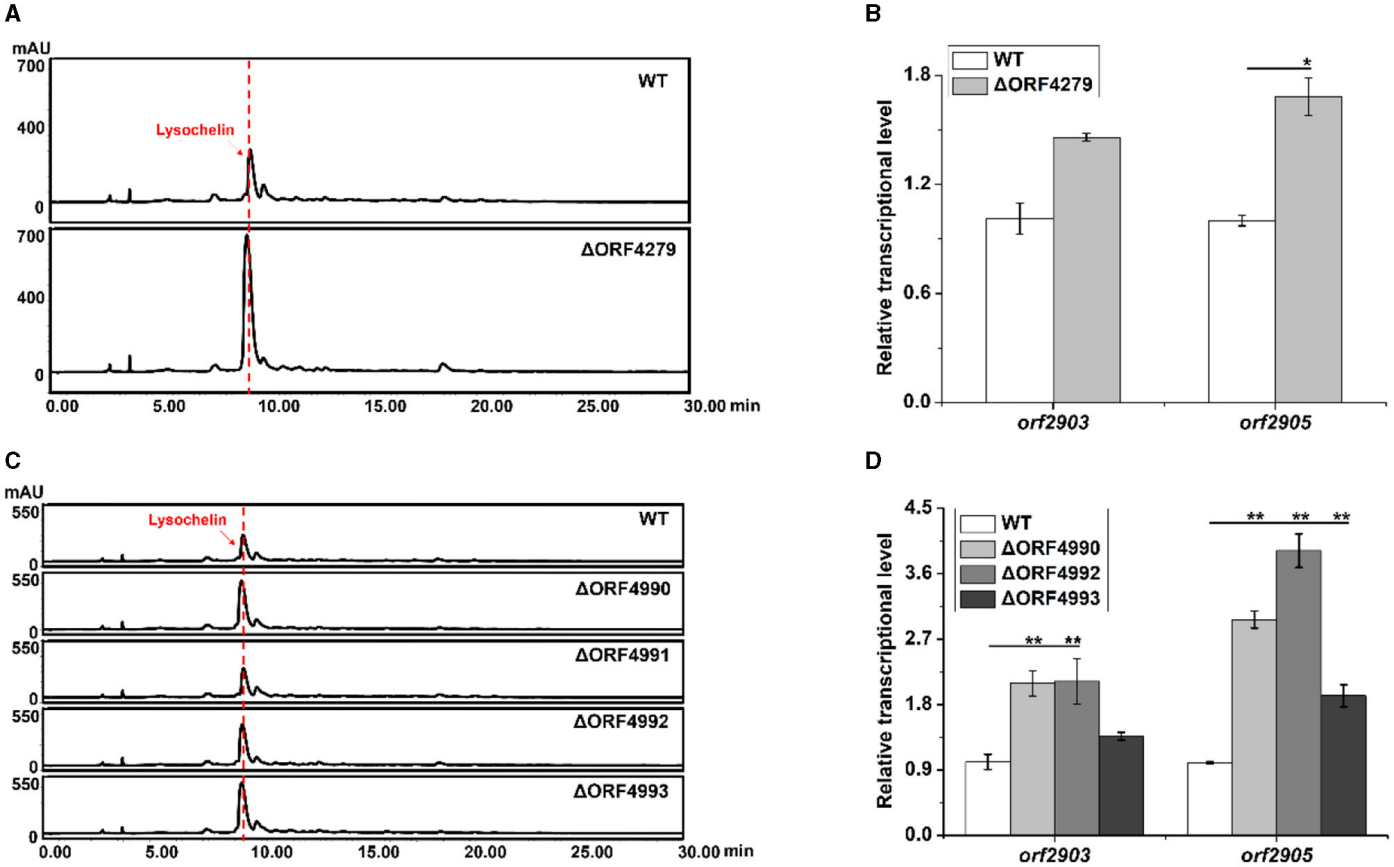
Effects of iron-utilizing genes on lysochelin biosynthesis. **(A)** HPLC analysis of lysochelin production in WT and the deletion mutant of SUF system regulator (ΔORF4279) grown in LBM medium for 72 h. **(B)** Transcriptional analysis of lysochelin biosynthetic genes (*orf2903* and *orf2905*) in WT (white columns) and ΔORF4279 (light-gray columns) grown in LBM for 24 h. **(C)** HPLC analysis of lysochelin production in WT and the deletion mutants of RpoE family RNA polymerase sigma factor (ΔORF4990), FecR family regulator (ΔORF4991), and bacterial transferrin receptors (ΔORF4992 and ΔORF4993) grown in LBM for 72 h. **(D)** Transcriptional analysis of *orf2903* and *orf2905* in WT (white columns), ΔORF4990 (light-gray columns), ΔORF4992 (gray columns), and ΔORF4993 (dark-gray columns) grown in LBM for 24 h. *p < 0.05; **p < 0.01.

With the finding that strain 3655 massively produced lysochelin only when reached a high cell density (Figure 3A), we wanted to explore potential regulatory mechanisms underlying the cell-density-dependent lysochelin biosynthesis. We performed the transcriptomics analysis of stain 3655 cultured to different densities, including OD_600_ 5.0 (DS group, lysochelin-deficiency) and OD_600_ 15.0 (S group, lysochelin-producing). In total, we obtained 23.07 and 16.84 million clean reads from DS group and S group, respectively. Among them, 1,476 differentially expressed genes (DEGs) were identified, including 347 up- and 1,476 downregulated genes, 79 DEGs specifically expressed in S group and 33 DEGs specifically expressed in DS group (Supplementary Figure 15). Expectedly, some interesting genes were upregulated accompany with lysochelin biosynthesis. The transcriptional level of *orf4279*, encoding SUF system Fe-S cluster assembly regulator, increased 4-fold in S group (Supplementary Tables 4, 5). qRT-PCR result showed the transcriptional level of *orf4279* was enhanced 24-fold at the OD_600_ value of 12.0 when compared to that of the OD_600_ value of 1.0, implying an important role in high cell density (Supplementary Figure 16A). SUF system is responsible for iron-sulfur cluster (Fe-S) biogenesis through assembly free iron and sulfides, which is crucial in bacteria for cell survival under stress conditions such as oxidation and iron starvation (Bai et al., 2018). Subsequently, we constructed the deletion mutant of *orf4279* (Supplementary Figure 13) and found that the mutant ΔORF4279 could produce more lysochelin, with the yield increased by 2.5-fold when compared to that of WT, and the transcriptional level of *orf2903* and *orf2905* in strain ΔORF4279 increased as well (Figures 5A, B and Supplementary Figure 17A). However, the cell growth of strain ΔORF4279 slightly decreased when compared to that of WT (Supplementary Figure 17B). This seems to suggest that the inhibition of SUF system might “trick” strain ΔORF4279 to “believe” the presence of a low iron condition and, therefore, more lysochelin biosynthesis was needed to grab iron from surroundings.

Moreover, the transcription of five clustered genes (*orf4990*-*orf4994*) was significantly upregulated in S group (Supplementary Table 4). The gene *orf4990* encodes RpoE family RNA polymerase sigma factor which may function in iron-restricted condition (Karash et al., 2022). The product of *orf4991* belongs to FecR family regulator that is related to regulation of ferric citrate uptake (Yokoyama et al., 2021). The gene *orf4992* encodes TonB-dependent transporter, and *orf4993* encodes Slam-dependent surface lipoprotein (SLP) which is homologous to transferrin-binding B (TbpB). These two proteins consist of bipartite transferrin receptor to acquire iron from transferrin (Pogoutse and Moraes, 2020). The product of *orf4994* belongs to the family of surface lipoprotein assembly modulators that are essential for surface display of SLPs (Hooda et al., 2017). To investigate the effect of these genes on lysochelin production, we generated the deletion mutants for each of these genes (Supplementary Figures 10, 13). Except *orf4991*, the deletion of *orf4990, orf4992*, or *orf4993* improved the yield of lysochelin, as well as the transcriptional levels of *orf2903* and *orf2905* (Figures 5C, D and Supplementary Figure 17C). However, the cell density of these mutants was similar to that of WT, with slightly increased in mutant ΔORF4993 (Supplementary Figure 17D). Besides, the transcriptional level of *orf4990, orf4992*, and *orf4993* was enhanced by 11-fold, 215-fold, and 13,005-fold at the OD_600_ 12.0 when compared to that of the OD_600_ 1.0 (Supplementary Figure 16). The data are consistent with the notion that the block of other iron acquisition pathways can “force” the mutants to produce more lysochelin to compete for iron.

### A TetR regulator regulated the biosynthesis of lysochelin

**Figure 6:**
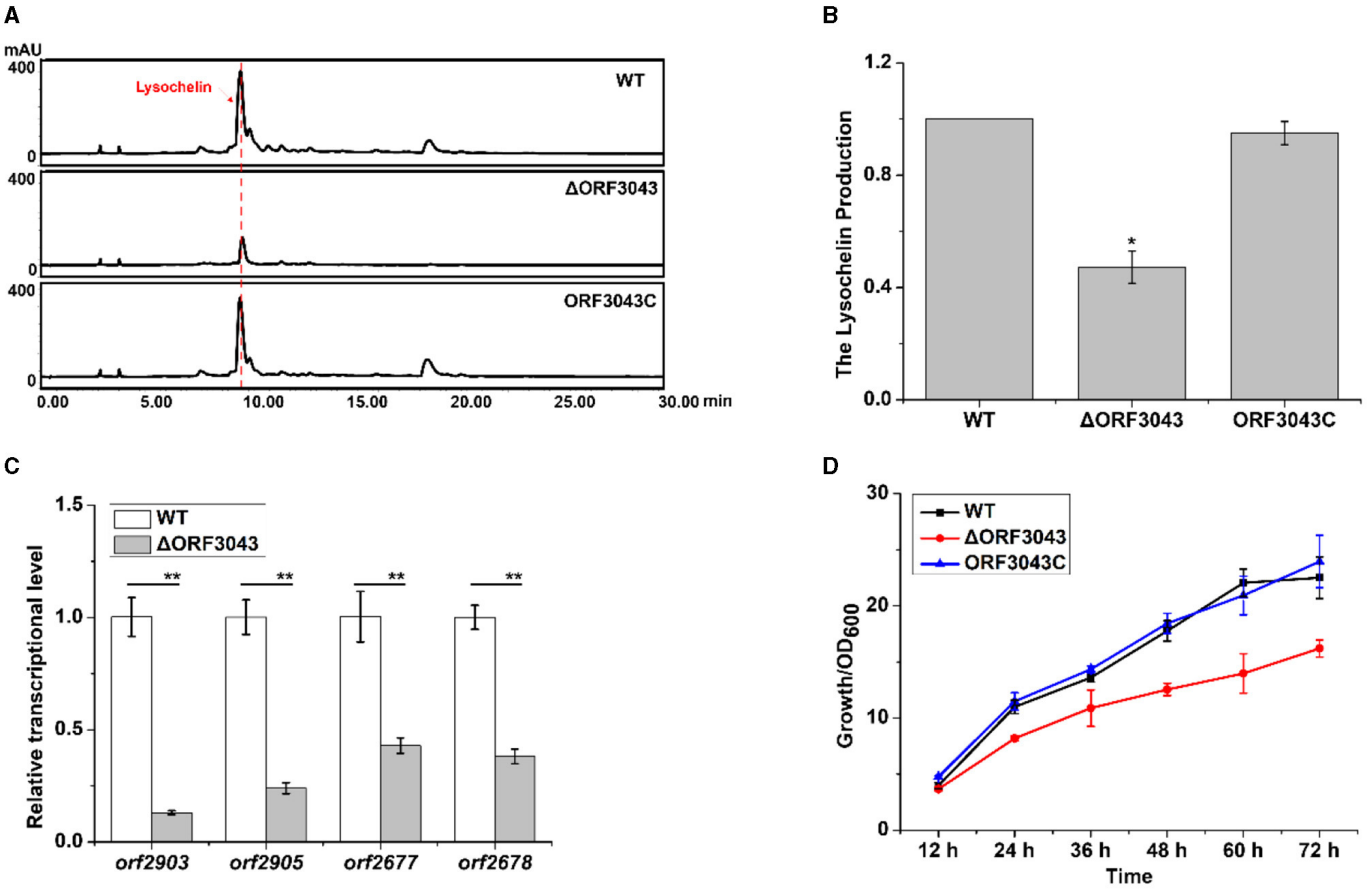
Regulation of lysochelin production by the TetR regulator ORF3043. **(A)** HPLC analysis of lysochelin production in WT, ΔORF3043 and its complementary strain ORF3043C grown in LBM medium for 72 h. **(B)** Quantification of lysochelin in the cultures from **(A)**. **(C)** Transcriptional analysis of *orf2903, orf2905, orf2677*, and *orf2678* in WT (white columns) and ΔORF3043 (light-gray columns) grown in LBM for 24 h. **(D)** The growth curve of WT (black line), ΔORF3043 (red line), and ORF3043C (blue line) grown in LBM medium. *p < 0.05; **p < 0.01.

In search for potential regulators, we found that the gene *orf3043* affected the biosynthesis of lysochelin. The product of this gene belongs to the TetR family of transcriptional regulators. We constructed the deletion mutant of *orf3043* (Supplementary Figure 18) and found that the transcriptional level of *orf2903* and *orf2905* in mutant ΔORF3043 was significantly decreased when compared to that of WT (Figure 6C). Accordingly, the yield of lysochelin reduced to 50% of that in WT (Figures 6A, B). Moreover, the deletion of *orf3043* reduced the transcriptional level of the maltose uptake genes *orf2677* and *orf2678*, indicating that the TetR might regulate maltose transportation (Figure 6C). The transcriptional level of *orf3043* was enhanced by 5-fold at the OD_600_ 12.0 when compared to that of the OD_600_ 1.0 (Supplementary Figure 16A). Next, we generated a complementary strain (ORF3043C) by reintroducing *orf3043* into the mutant (Supplementary Figure 18). As expected, the production of lysochelin in the complementary strain was restored to the WT level (Figures 6A, B). While the cell density of the mutant ΔORF3043 decreased to 70% of that in WT, the density was restored to the WT level in the complementary strain ORF3043C (Figure 6D). The results indicated that the TetR regulator ORF3043 contributes to the regulation of lysochelin biosynthesis through affecting the transcription of maltose transportation genes and then lysochelin biosynthetic genes.

## Discussion

In this work, we found maltose dramatically induced the production of a compound in *Lysobacter* strain 3655 cultured in the rich medium LB. The results from gene deletion of the *lec*-like cluster and LC-MS analysis proved this compound to be lysochelin, the first siderophore isolated from *Lysobacter* (Miller et al., 2023). The production of lysochelin depended on the growth status of strain 3655 cultured in LBM medium. At low cell density (represented as the OD_600_ value of 5.0), the siderophore biosynthesis was completely inhibited in the rich medium, and lysochelin production was observed when the OD_600_ reached 7.5, and then significantly increased at high cell density (OD_600_ 15.0). Strain 3655 grew much better in LBM than that in LB medium; in LBM the OD_600_ value could reach nearly 20.0. The high cell growth led to gradual depletion of iron in LBM medium. The data indicated that, when the OD_600_ value reached 7.5, the iron concentration was lowered to the threshold to trigger lysochelin production. As the cell density continued to increase, lysochelin was massively produced. Conversely, in the absence of maltose, strain 3655 grew in LB medium to a maximum OD_600_ value of 6.0, and iron was not depleted to the threshold concentration, thus no lysochelin was observed. Maltose could also promote the high-density growth and induce lysochelin biosynthesis in *L. enzymogenes* OH11 (Supplementary Figure 1), indicating that this might be a general phenomenon in *Lysobacter*, in which siderophore production can be induced through promoting the high-density growth.

The transportation of maltose into cells is the initiation of maltose metabolism. In *E. coli*, the periplasmic binding protein-dependent ATP binding cassette (ABC) transporter is responsible for maltose uptake, which is encoded by *malEFGK* genes (Boos and Shuman, 1998). We found strain 3655 contains the *mal-*like genes, *orf2677, orf2678, orf2679*, and *orf2685* that are homologous to *malE, malF, malG*, and *malK*, respectively (Supplementary Figures 7, 8, 19, 20). The deletion of *orf2677* or *orf2678* impaired, but not completely inhibited, cell growth and lysochelin production, implying that other transport systems might be present in strain 3655. For example, the phosphotransferase system (PTS) is responsible for maltose transportation in *Enterococcus faecalis* (Breton et al., 2005). In *E. coli*, maltose is degraded into glucose and glucose-1-phosphate by three enzymes MalQ (amylomaltase), MalP (maltodextrin phosphorylase), and MalZ (maltodextrin glucosidase). Glucose and glucose-1-phosphate are then converted to glucose-6-phosphate, which enter in the glycolysis pathway with glucokinase and phosphoglucomutase, respectively (Boos and Shuman, 1998). However, the homologous genes of *malQ, malP*, and *malZ* were not found in strain 3655. In *Streptococcus bovis*, maltose is cleaved by a maltose hydrolase into two glucose molecules (Martin and Russell, 1987; Andersson and Rådström, 2002). Moreover, the maltose phosphorylase could convert maltose to glucose and glucose-1-phosphate in *Lactococcus lactis* (Levander et al., 2001; Nilsson and Rådström, 2001). In strain 3655, we found *orf2742* was homologous to genes encoding maltose hydrolase (possible phosphorylase). Surprisingly, the deletion of *orf2742* did not affect the cell growth and lysochelin production. Further studies are needed to explore if strain 3655 could contain other uncharacterized maltose metabolism enzymes.

The lysochelin biosynthesis was induced at high cell density in strain 3655, and the deletion of *orf644*, which encodes a dioxygenase for the biosynthesis of the quorum sensing (QS) signal DF, significantly decreased the cell growth and lysochelin production. The transcription of the lysochelin biosynthetic genes (*orf2903* and *orf2905*) and the maltose transportation genes (*orf2677* and *orf2678*) was clearly reduced in mutant ΔORF644. The results suggested that the DF signal system might regulate maltose transportation and then control lysochelin biosynthesis. The quorum sensing might also be related to the interesting observation, in which the culture volumes could make a big difference in cell density and lysochelin production even if the same concentration of maltose was used for cultures. More studies are needed to unveil the molecular details behind this phenomenon. The QS regulation of siderophores is present in many microbes. The yield of siderophore pyoverdine was decreased by 2-fold in the mutant of *lasR* encoding QS regulator of *Pseudomonas aeruginosa* (Stintzi et al., 1998). The QS antagonist furanone could stimulate pyoverdine biosynthesis in *P. aeruginosa*, while inhibiting pyoverdine formation in *P. putida* (Ren et al., 2005). However, the lack of the QS regulator CviR led to an increase of siderophore production in *Chromobacterium violaceum* (Batista et al., 2024). The mechanism underlying the QS-regulated siderophore production is not fully understood, but could probably be due to the divergent survival strategies adopted by various species (McRose et al., 2018). Recently, it was found that the siderophore yersiniabactin of uropathogenic *Escherichia coli* also functions as a QS signal, which represents a convergence of the quorum-sensing and the siderophore system (Heffernan et al., 2024). In *Vibrio harveyi*, QS regulates two types of siderophores in different environmental and growth contexts, producing cell-associated siderophores at low cell density and accumulating soluble siderophores at high cell density (McRose et al., 2018).

The transcriptomics analysis and qRT-PCR results showed the transcriptional level of bacterial transferrin receptor genes (*orf4992* and *orf4993*) was greatly enhanced at high cell density under iron starvation conditions. This receptor has been found in some members of the *Betaproteobacteria* and *Gammaproteobacteria* and is vital for survival of several important human and animal pathogens (Pogoutse and Moraes, 2020). The receptor consists of two proteins including TonB-dependent transporter (TbpA) and surface lipoprotein (TbpB), which binds transferrin and then removes iron from the C-terminal lobe of transferrin and transports it across the bacterial outer membrane (Pogoutse and Moraes, 2017). Therefore, this receptor is responsible for iron acquisition from transferrin, which is an iron-carrying glycoprotein found in the mammalian circulatory system (Hughes and Friedman, 2014). This receptor is essential for colonizing and invading pathogens, such as the families of *Neisseriaceae, Pasteurellaceae*, and *Moraxellaceae*, to obtain iron from their hosts (Pogoutse and Moraes, 2017). The *Lysobacter* genus belongs to *Gammaproteobacteria*. Although they are not known to be pathogenic bacteria, they could prey on other bacteria, fungi, algae, and nematodes (Christensen and Cook, 1978; Yue et al., 2021). The presence of bacterial transferrin receptor might confer the survival advantage on *Lysobacter*, since the transferrin homologs are also found in invertebrates and algae (Lambert, 2012). Apart from lysochelin, strain 3655 activated bacterial transferrin receptor to enhance its ability for iron acquisition under iron restriction environment, thus improved the survivability.

Additionally, we found that the TetR family regulator ORF3043 controlled the cell growth and lysochelin production through the transcriptional regulation of maltose transportation genes (*orf2677* and *orf2678*) and lysochelin biosynthetic genes (*orf2903* and *orf2905*). The transcriptional level of itself was significantly enhanced at high cell density, which showed ORF3043 might function under high-density growth of strain 3655. TetR regulators have been proved to regulate siderophore production in various bacteria. A global regulator LuxT activates siderophore production via inhibiting the expression of a repressor SwrZ at low cell density of *Vibrio harveyi* (Eickhoff et al., 2022). In the rhizobacterium *Pseudomonas fluorescens*, the TetR regulator PhlH represses the expression of proteins involved in pyoverdine biosynthesis based on comparative proteomic analysis (Zhang et al., 2022). The pathway-specific regulator Orf12 from the biosynthetic cluster of 7-hydroxytropolone (7-HT) positively regulates 7-HT production through stimulating the expression of orf6-orf9 in the 7-HT cluster of *Pseudomonas donghuensis* HYS (Chen et al., 2018).

In summary, we found that maltose induced lysochelin production through promoting the high-density growth of *Lysobacter* sp. 3655, which resulted in an iron-restricted condition that activated the expression of the siderophore biosynthetic genes even when the bacterium was grown in rich media. Through generating a series of gene deletion mutants and analyzing the gene transcription and siderophore production, we showed that maltose metabolism and quorum sensing were involved in the siderophore biosynthesis in rich media. Furthermore, strain 3655 employed both the siderophore and the transferrin receptor to grab the trace amount of extracellular iron. The data also showed that a TetR regulator positively regulated lysochelin production through affecting the maltose metabolism. The studies shed new light on the regulation of siderophore biosynthesis, which could provide a new way to siderophore production through high-density scale-up fermentation.

## Data availability statement

The datasets presented in this study can be found in online repositories. The names of the repository/repositories and accession number(s) can be found in the article/Supplementary material.

## Author contributions

FZ: Writing – review & editing, Investigation, Methodology. JL: Investigation, Methodology, Writing – review & editing. LJ: Investigation, Methodology, Writing – review & editing. YZ: Investigation, Methodology, Writing – review & editing. LY: Conceptualization, Data curation, Funding acquisition, Supervision, Writing – original draft, Writing – review & editing. LD: Conceptualization, Supervision, Writing – original draft, Formal analysis, Writing – review & editing.
