# Supplementary material for: Production of the siderophore lysochelin in rich media through maltose-promoted high-density growth of Lysobacter sp. 3655
Source: Front Microbiol. 2024 Jun 26;15:1433983. doi: 10.3389/fmicb.2024.1433983 (PMC11233812; doi:10.3389/fmicb.2024.1433983)
Supplement: Supplementary file 1 [file Data_Sheet_1.pdf]

Production of the Siderophore Lysochelin in Rich Media through Maltose-promoted High-density Growth of *Lysobacter* sp. 3655

Fang Zhang[1†], Jia Liu[1†], Lin Jiang[1], Yongbiao Zheng[1], Lingjun Yu[1*], Liangcheng Du[2*]

[1]School of Life Sciences, Fujian Normal University, Fuzhou, Fujian, China

[2]Department of Chemistry, University of Nebraska-Lincoln, Lincoln, NE, USA

***For corresponding:** Lingjun Yu, School of Life Sciences, Fujian Normal University, Fuzhou 350117, Fujian, China. E-mail: yulj113@fjnu.edu.cn. Liangcheng Du, Department of Chemistry, University of Nebraska-Lincoln, Lincoln, NE 68588, USA. E-mail: ldu3@unl.edu.

[†]These authors contribute equally to this work.

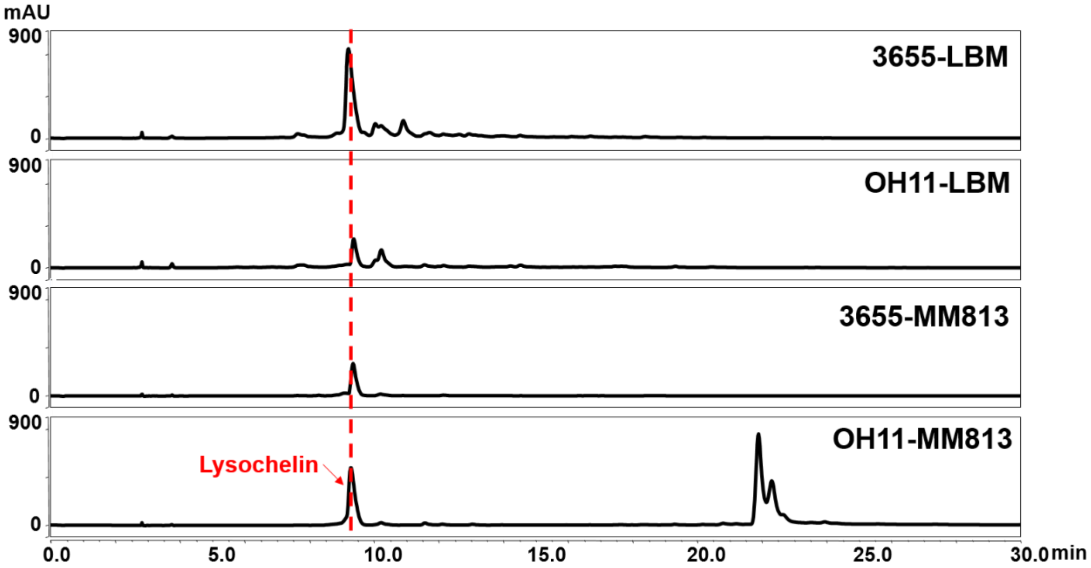

Figure S1. HPLC analysis of lysochelin production in *Lysobacter* sp. 3655 and *Lysobacter enzymogenes* OH11 grown in LBM or MM813 for 72 h. LBM, LB medium containing 5% (w/v) maltose; MM813, a modified M813 medium without $FeSO_4$.

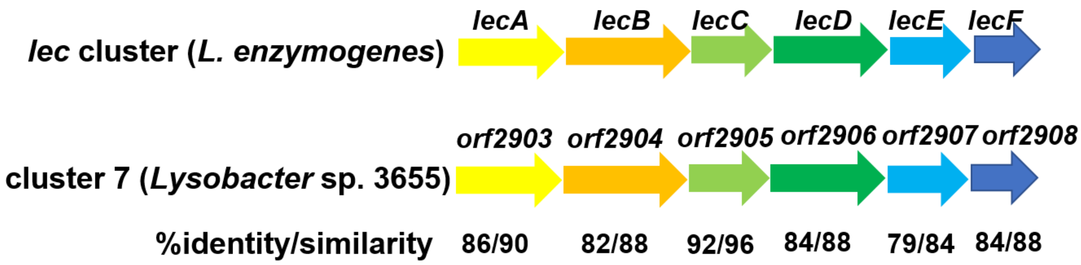

Figure S2. Comparison of the deduced amino acid sequences of the Cluster-7 genes (accession number PP824363) from *Lysobacter* sp. 3655 with that of the lysochelin gene cluster (*lec*) from *Lysobacter enzymogenes* OH11.

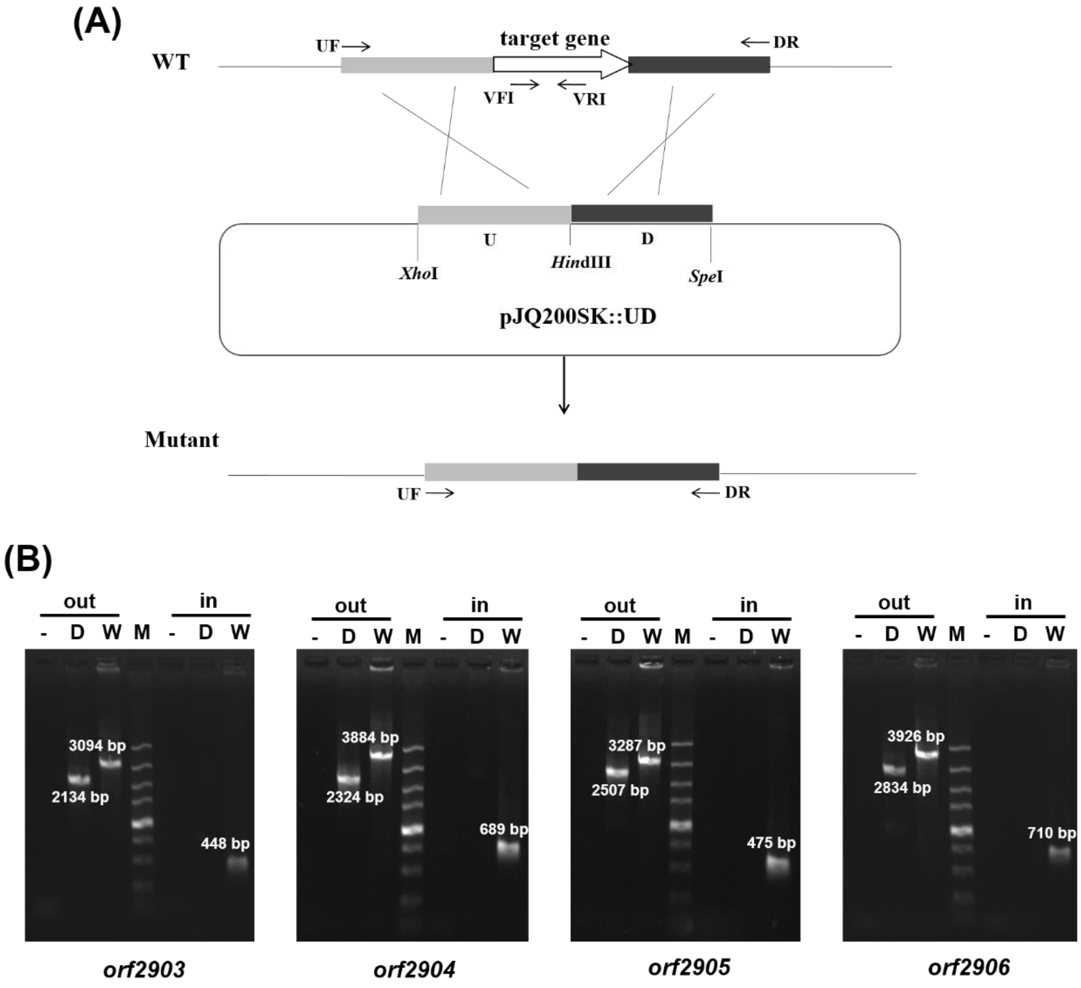

Figure S3. Generation of gene deletion mutants of *orf2903*, *orf2904*, *orf2905* and *orf2906*. (A) Schematic representation of the deletion mutants in strain 3655 (WT). (B) PCR verification of the mutant strains ΔORF2903, ΔORF2904, ΔORF2905, and ΔORF2906. -: negative control, H_2O used as the template; out-D: PCR products using UF/DR as the primers and genomic DNA of the corresponding deletion mutant as templates; out-W: positive control, PCR products using UF/DR as the primers and genomic DNA of WT as templates; in-D: PCR products using VFI/VRI as the primers and genomic DNA of the corresponding deletion mutant as templates; in-W: positive control, PCR products using VFI/VRI as the primers and genomic DNA of WT as templates; M: DNA marker. U, the upstream region of target gene; D, the downstream

region of target gene.

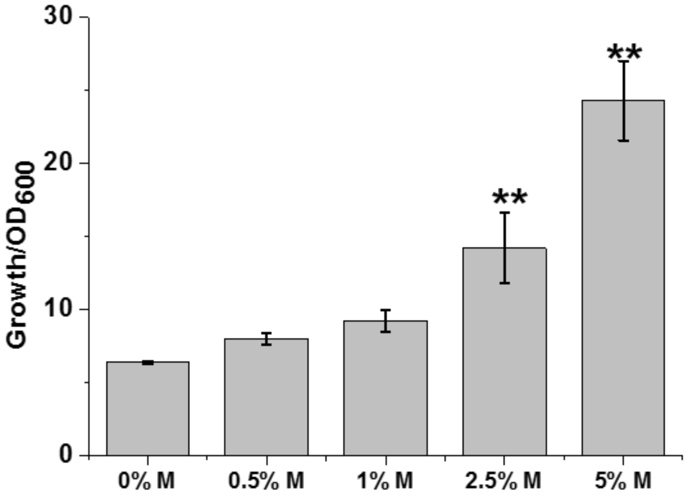

Figure S4. The cell density as represented by OD_{600} of WT cultured in LB medium with different amount of maltose (0%, 0.5%, 1%, 2.5%, and 5%) at 72 h. M, maltose; ** $p < 0.01$.

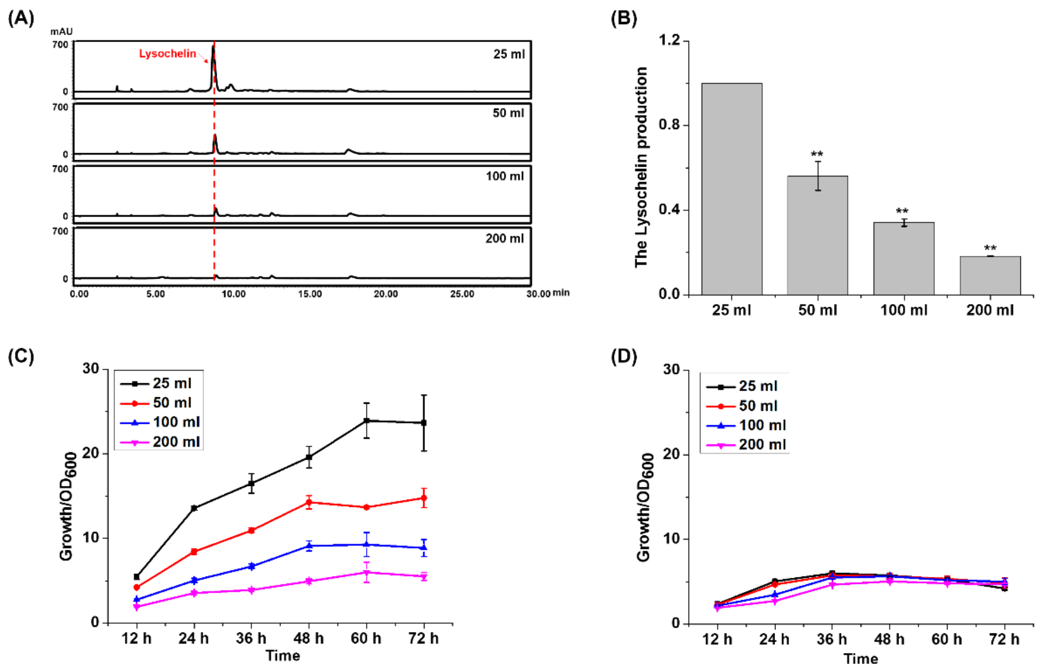

Figure S5. The effect of the cultural volume on lysochelin production. (A) HPLC analysis of lysochelin production in WT of different cultural volumes of LBM (25, 50, 100, and 200 ml) for 72 h. (B) Quantification of relative yield of lysochelin in these cultures, ** $p < 0.01$. (C) Growth curve of WT in different cultural volumes of LBM. (D) Growth curve of WT in different cultural volumes of LB.

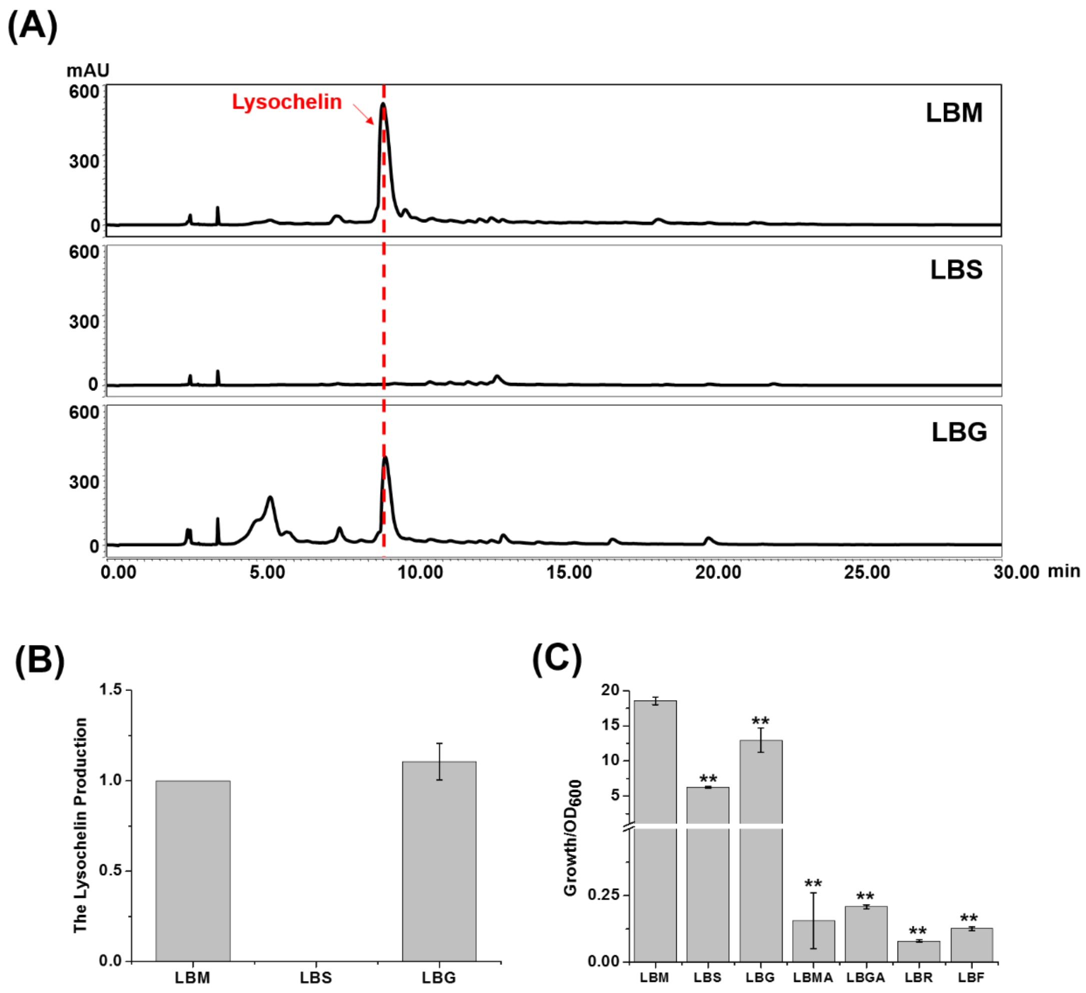

Figure S6. The effect of various carbohydrates on lysochelin production. (A) HPLC analysis of lysochelin production in WT cultured in LB with maltose (5%, LBM), sucrose (5%, LBS), or glucose (5%, LBG) for 72 h. (B) Quantification of relative yield of lysochelin in these cultures. (C) Growth of WT cultured in LB with different carbohydrates at 72 h. LBMA, LB with 1% mannose; LBGA, LB with 1% galactose; LBR, LB with 1% rhamnose; LBF, LB with 1% fucose; ** $p < 0.01$.

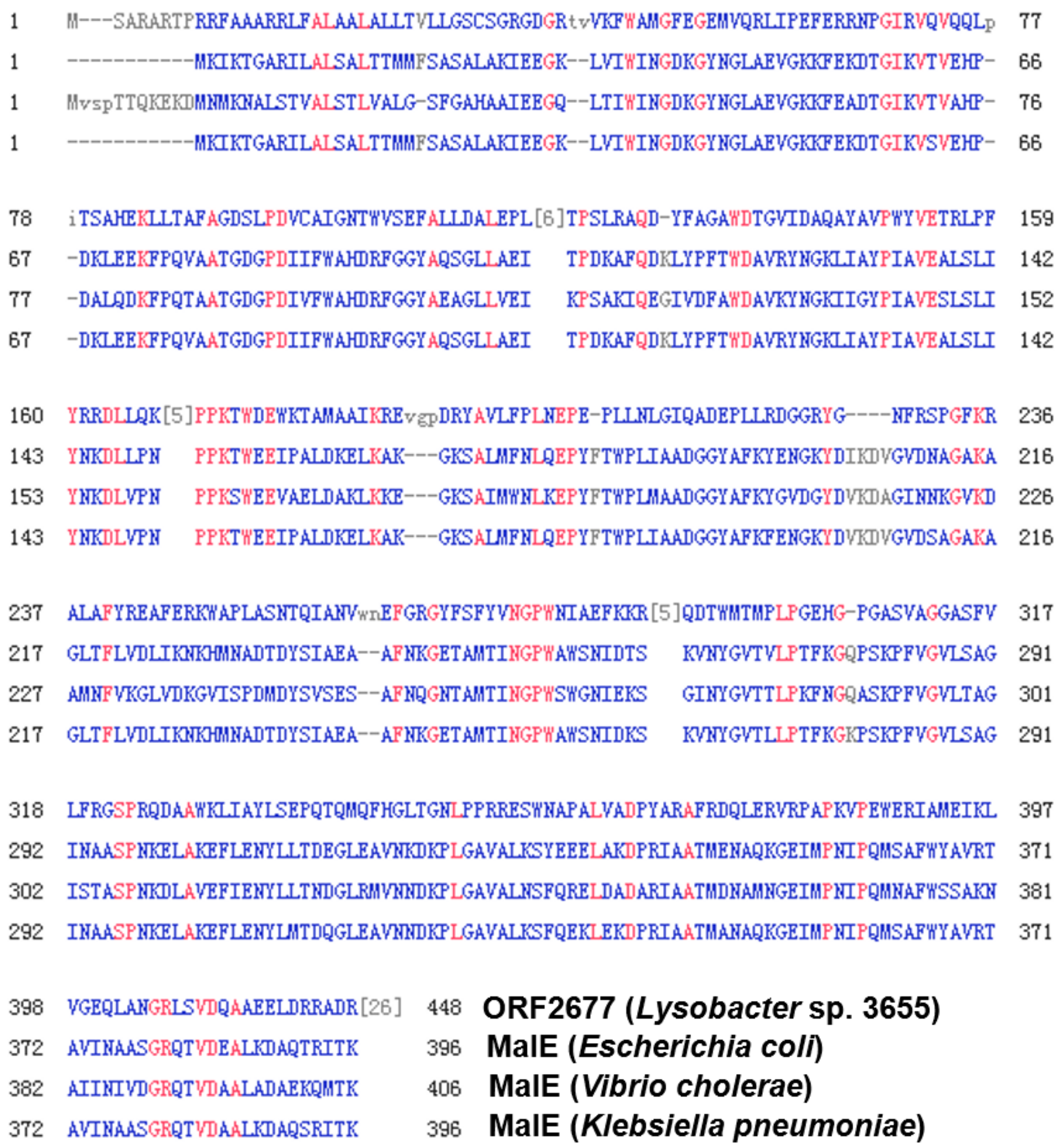

Figure S7. Alignment of the amino acid sequence of ORF2677 (accession number PP824351) in strain 3655 with MalE protein from *Escherichia coli*, *Vibrio cholerae*, and *Klebsiella pneumoniae*. The red color indicates the identical residues, and the blue indicates the highly conserved ones.

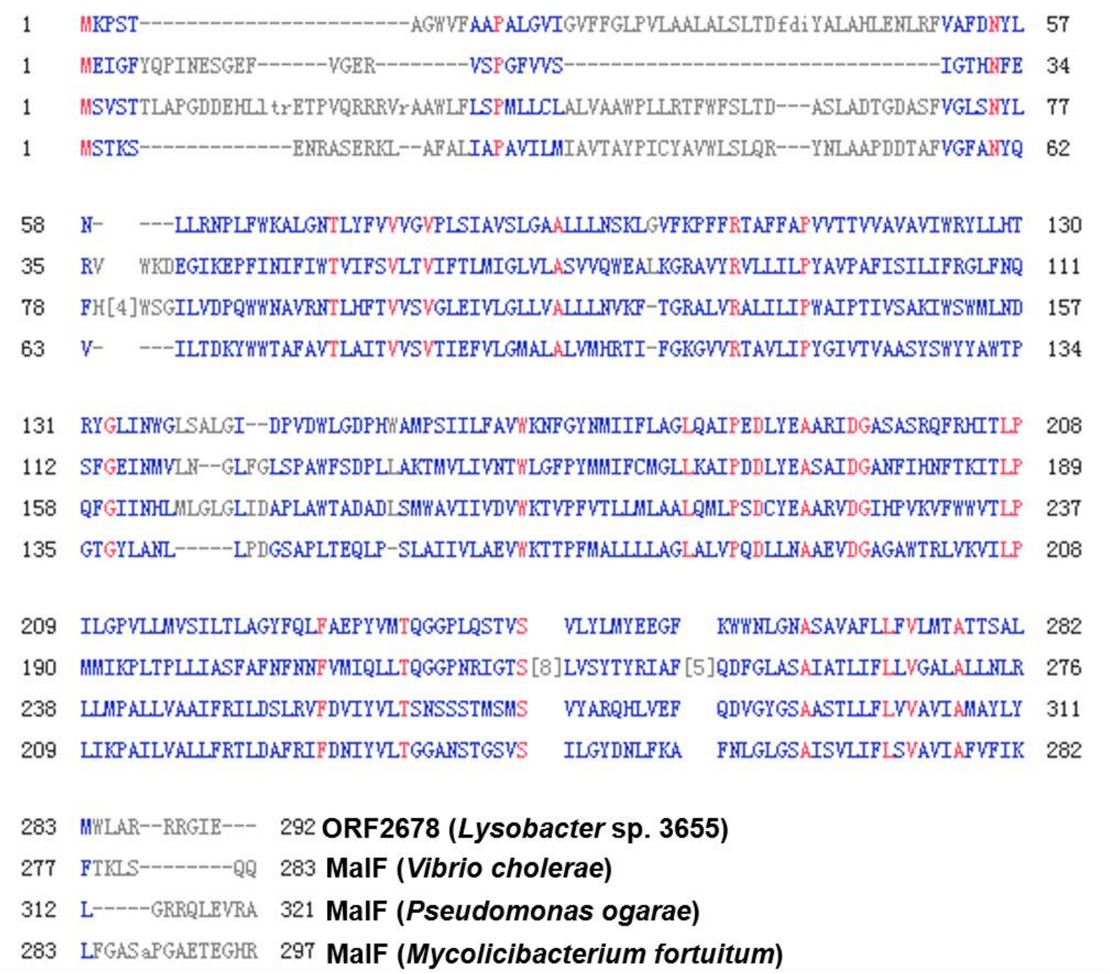

Figure S8. Alignment of the amino acid sequence of ORF2678 (accession number PP824352) in strain 3655 with MalF protein from *Vibrio cholerae*, *Pseudomonas ogarae*, and *Mycolicibacterium fortuitum*. The red color indicates the identical residues, and the blue indicates the highly conserved ones.

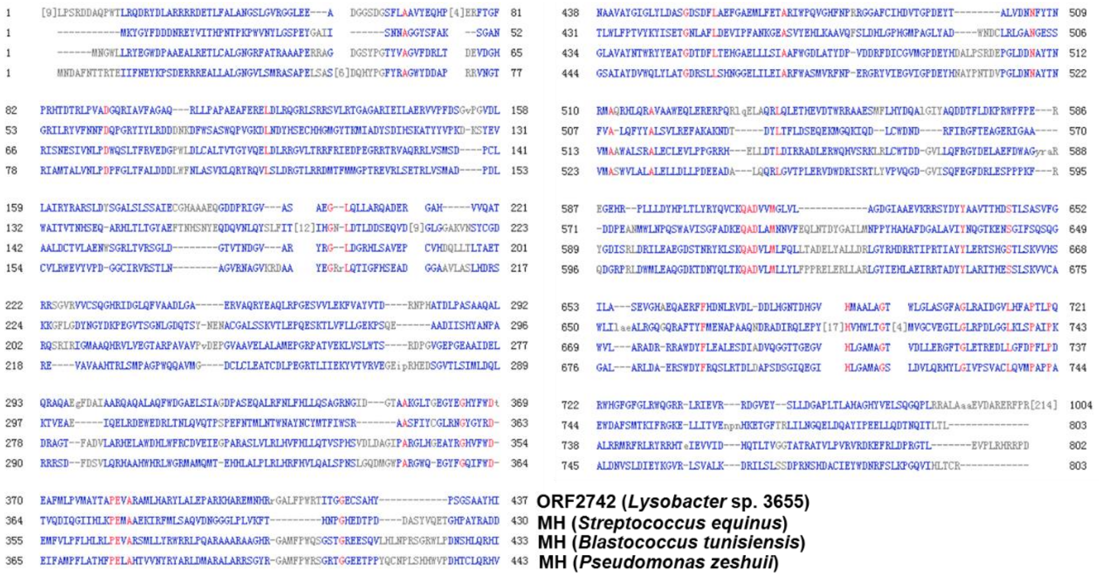

Figure S9. Alignment of the amino acid sequence of ORF2742 (accession number PP824353) in strain 3655 with maltose hydrolase (MH) from *Streptococcus equinus*, *Blastococcus tunisiensis*, and *Pseudomonas zeshuii*. The red color indicates the identical residues, and the blue indicates the highly conserved ones.

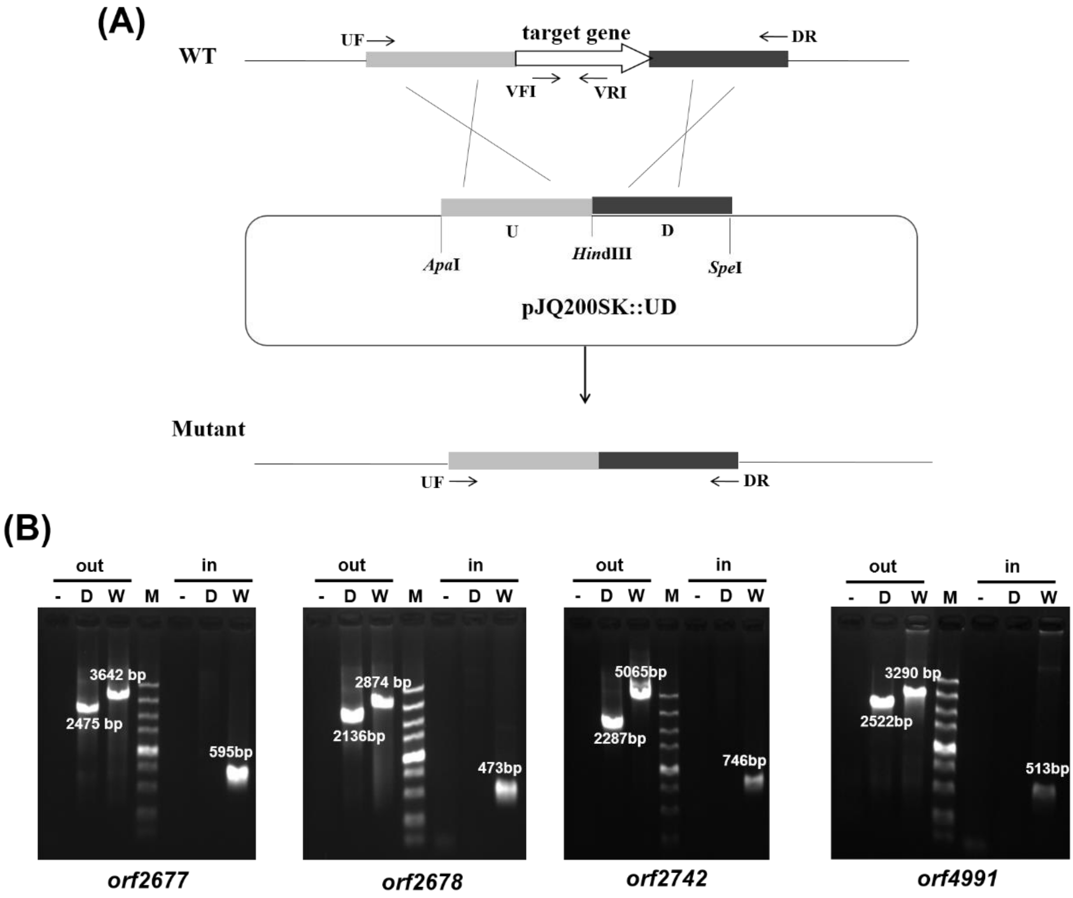

Figure S10. Generation of gene deletion mutants of *orf2677*, *orf2678*, *orf2742*, and *orf4991* (accession number PP824357). (A) Schematic representation of the deletion mutants in strain 3655 (WT). (B) PCR verification of the mutant strains ΔORF2677, ΔORF2678, ΔORF2742, and ΔORF4991. -: negative control, H_2O used as the template; out-D: PCR products using UF/DR as the primers and genomic DNA of the corresponding deletion mutant as templates; out-W: positive control, PCR products using UF/DR as the primers and genomic DNA of WT as templates; in-D: PCR products using VFI/VRI as the primers and genomic DNA of the corresponding deletion mutant as templates; in-W: positive control, PCR products using VFI/VRI as the primers and genomic DNA of WT as templates; M: DNA marker. U, the upstream region of target gene; D, the downstream region of target gene.

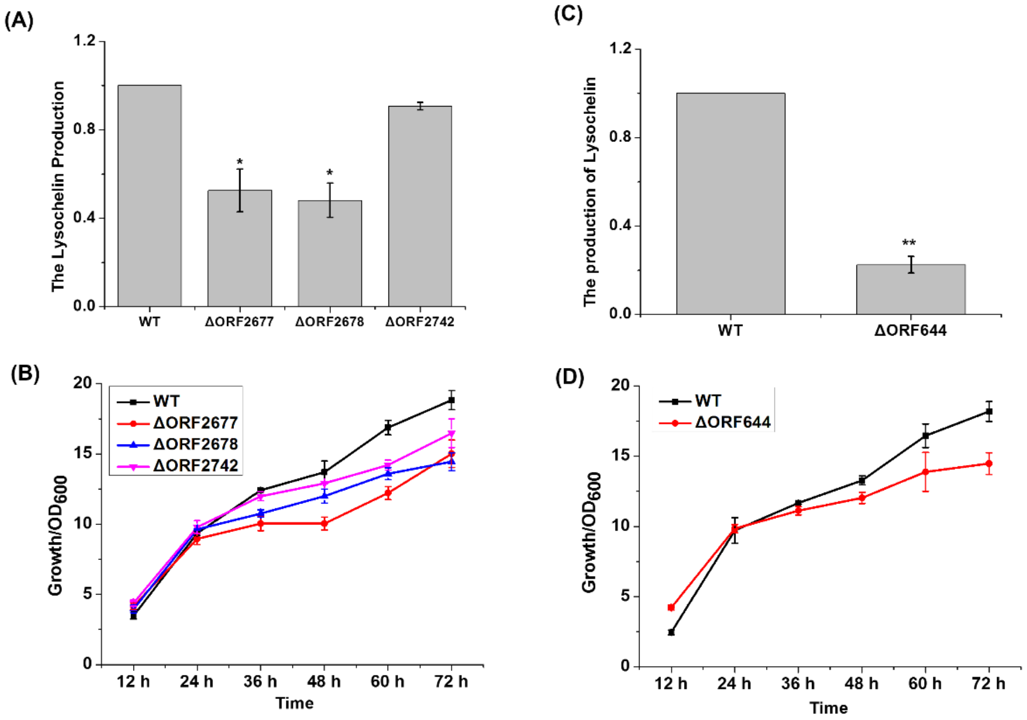

Figure S11. The growth and lysochelin production in WT and the deletion mutants. (A) Quantification of relative yield of lysochelin in WT, ΔORF2677, ΔORF2678, and ΔORF2742 cultured in LBM for 72 h. (B) The growth curve of WT, ΔORF2677, ΔORF2678, and ΔORF2742 cultured in LBM. (C) Quantification of relative yield of lysochelin in WT and ΔORF644 cultured in LBM for 72 h. (D) The growth curve of WT and ΔORF644 cultured in LBM. * $p < 0.05$; ** $p < 0.01$.

```
MSAAPAMVGGPRLQVDYLDADPAQLLAEDDTLALFGFGQDAPHHDDPRYLRVPLEPYGDR  60
MSAA A V GPRL VDYLD DPAQ LA+DDTLALFGFG+DAPHHDDPRYLRVPLEP GDR
MSAAAATVSGPRLDVDYLDTDPAQALAQDDTLALFGFGRDAPHHDDPRYLRVPLEPLGDR  60

RLERWRGLGPVASGRDGDLAWSQDGTLQFGVIEIDEPEPLPGEPVNGEIGRAAEYLYRKL  120
RLERWRG GPVA GRDGDLAW++DG LQFGVIE+DEP PLPGE  NGEIGRAAEYLYRKL
RLERWRGNGPVARGRDGDLAWAEDGALQFGVIELDEPAPLPGESANGEIGRAAEYLYRKL  120

LAFTAQRGYPHLLRIWNYLDGITLGHGDEERYRVFCVGRAAGLGEFPIARLPAATAIGRV  180
LAFTAQRGYP LLRIWNYLDGITLGHGDEERYRVFCVGRAAGLGEFPIARLPAATAIGRV
LAFTAQRGYPQLLRIWNYLDGITLGHGDEERYRVFCVGRAAGLGEFPIARLPAATAIGRV  180

DGARRLQVYWLASRVPGTPLENPRQVSAYRYPRRYGPQSPSFARAMLPPQPAQMPLLLSG  240
DGARRLQVYWLASR PGTPLENPRQVSAYRYPR+YGPQSPSFARAMLPP PA+MPLLLSG
DGARRLQVYWLASRTPGTPLENPRQVSAYRYPRQYGPQSPSFARAMLPPGPARMPLLLSG  240

TAAVVGHESRHADSVLAQLEETLANFDSLIGAARQQRPGLPAAFGPGSRLKVYVRDLPDL  300
TAAVVGHESRHA+SVLAQLEETLANFDSLIGAARQ RP LP AFGPGSRLKVYVRDL DL
TAAVVGHESRHAESVLAQLEETLANFDSLIGAARQHRPDLPPAFGPGSRLKVYVRDLHDL  300

PLLQAELDRRYGDRVPRVLLHAAVCRHELLVEIDGVHG  338 ORF644 (Lysobacter sp. 3655)
P++ AELDRRYGDRVPR+L+HAAVCRHELLVEIDGVHG       Consensus sequence
PIVAAELDRRYGDRVPRILVHAAVCRHELLVEIDGVHG  338 LenB2 (Lysobacter enzymogenes OH11)
```

Figure S12. Alignment of the amino acid sequence of ORF644 (accession number PP824350) in strain 3655 with LenB2 protein from *L. enzymogenes* OH11.

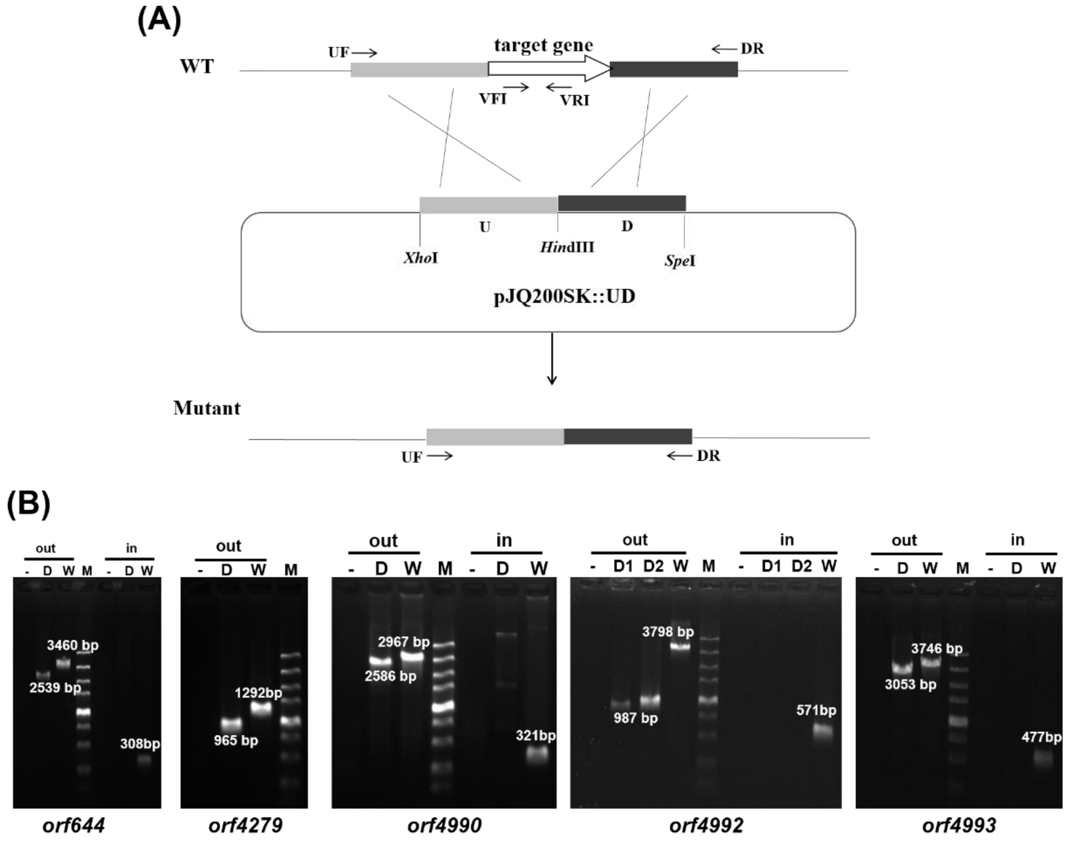

Figure S13. Generation of gene deletion mutants of *orf644* (PP824350), *orf4279* (PP824355), *orf4990* (PP824356), *orf4992* (PP824358), and *orf4993* (PP824359). (A) Schematic representation of the deletion mutants in strain 3655 (WT). (B) PCR verification of the mutant strains ΔORF644, ΔORF4279, ΔORF4990, ΔORF4992, and ΔORF4993. -: negative control, H_2O used as the template; out-D: PCR products using UF/DR as the primers and genomic DNA of the corresponding deletion mutant as templates; out-W: positive control, PCR products using UF/DR as the primers and genomic DNA of WT as templates; in-D: PCR products using VFI/VRI as the primers and genomic DNA of the corresponding deletion mutant as templates; in-W: positive control, PCR products using VFI/VRI as the primers and genomic DNA of WT as templates; M: DNA marker. U, the upstream region of target gene; D, the downstream region of target gene.

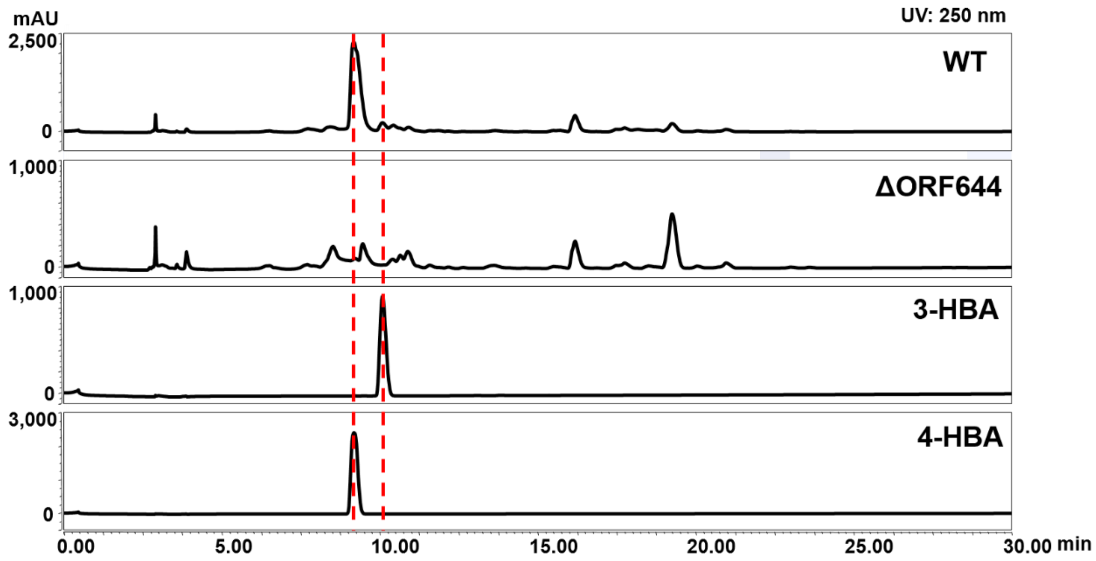

Figure S14. HPLC analysis of 3-hydroxybenzoic acid (3-HBA) and 4-hydroxybenzoic acid (4-HBA) production in WT and ΔORF644 cultured in LBM for 72 h, using a detector with the wavelength of 250 nm. Standard 3-HBA and 4-HBA were included as references.

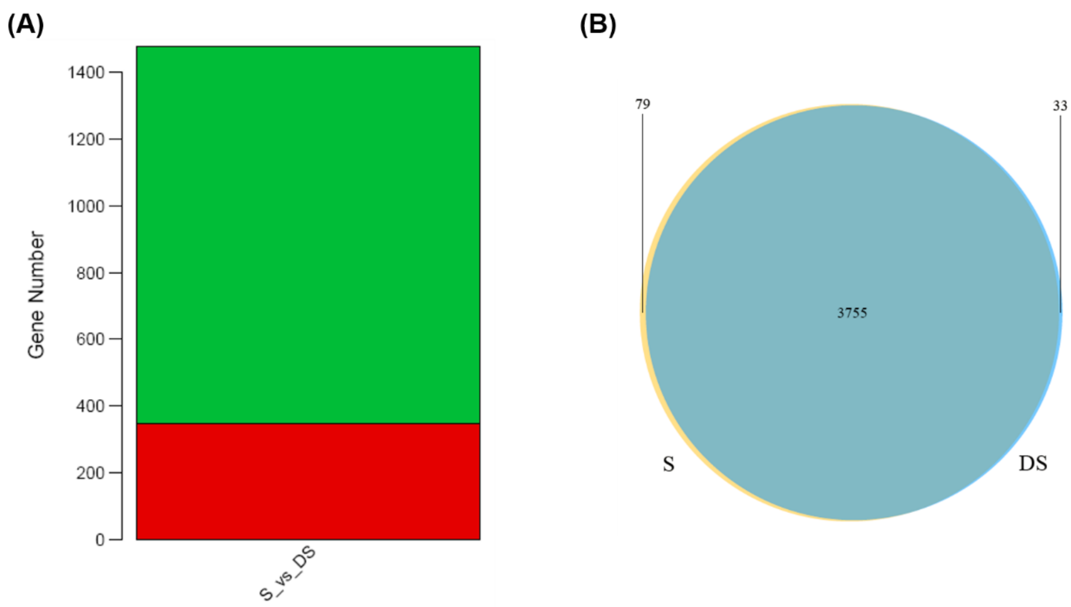

Figure S15. The differentially expressed genes (DEGs) between the DS group and the S group. (A) The barplot of DEGs, with red barplot representing upregulated genes and the green barplot representing downregulated genes; (B) The venn map of gene expression, with the overlapping area representing genes expression common for both groups, the non-overlapping area representing genes expression specific for each group. DS, sample of low cell density culture that did not produce lysochelin; S, sample of high cell density culture that produced lysochelin.

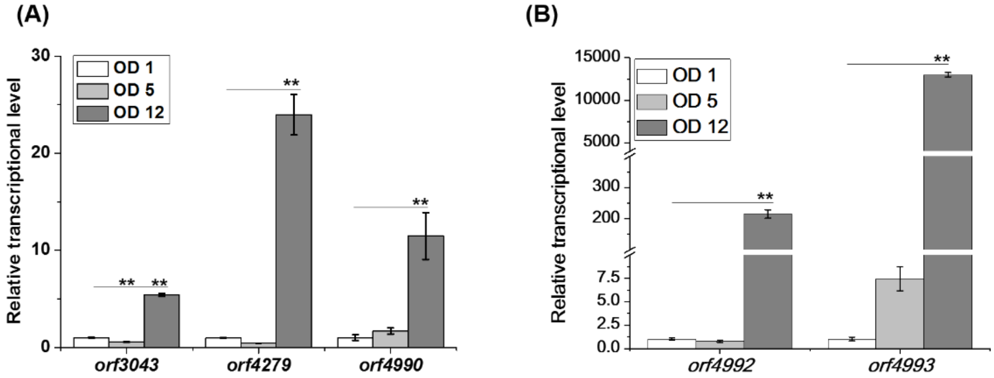

Figure S16. Transcription analysis of several regulator genes in WT at different cell densities. (A) Relative transcriptional level of *orf3043* (PP824354) encoding a TetR transcriptional regulator gene, *orf4279* (PP824355) encoding a SUF system Fe-S cluster assembly regulator, and *orf4990* (PP824356) encoding a RpoE family RNA polymerase sigma factor. (B) Relative transcriptional level of the genes *orf4992* (PP824358) and *orf4993* (PP824359) encoding the bipartite transferrin receptors. The WT cultures were grown in LBM and analyzed at the OD_{600} value of 1 (white columns), 5 (light-grey columns), and 12 (grey columns). ** $p < 0.01$

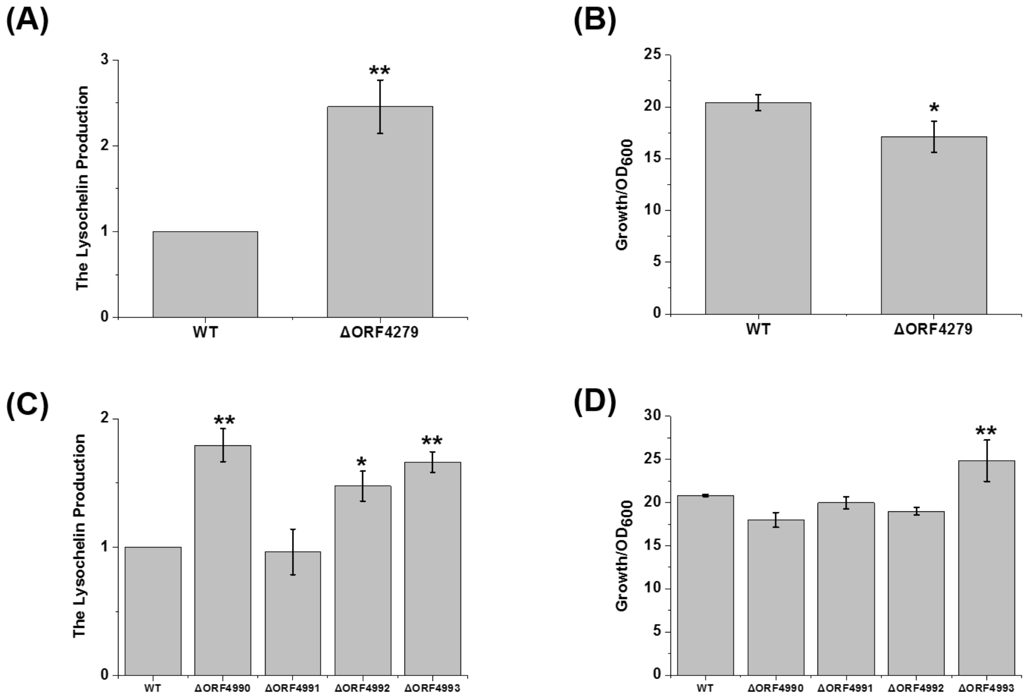

Figure S17. The growth and lysochelin production in WT and deletion mutants of regulator genes. (A) Quantification of relative yield of lysochelin in WT and the SUF system regulator mutant ΔORF4279 cultured in LBM for 72 h. (B) Cell density of WT and ΔORF2742 cultured in LBM at 72 h. (C) Quantification of relative yield of lysochelin in WT, mutant of RpoE family RNA polymerase sigma factor gene ΔORF4990, mutant of FecR family regulator gene ΔORF4991, and the mutants of bacterial transferrin receptor genes ΔORF4992 and ΔORF4993, cultured in LBM for 72 h. (D) Cell density of WT, ΔORF4990, ΔORF4991, ΔORF4992, and ΔORF4993 cultured in LBM at 72 h. * $p < 0.05$; ** $p < 0.01$.

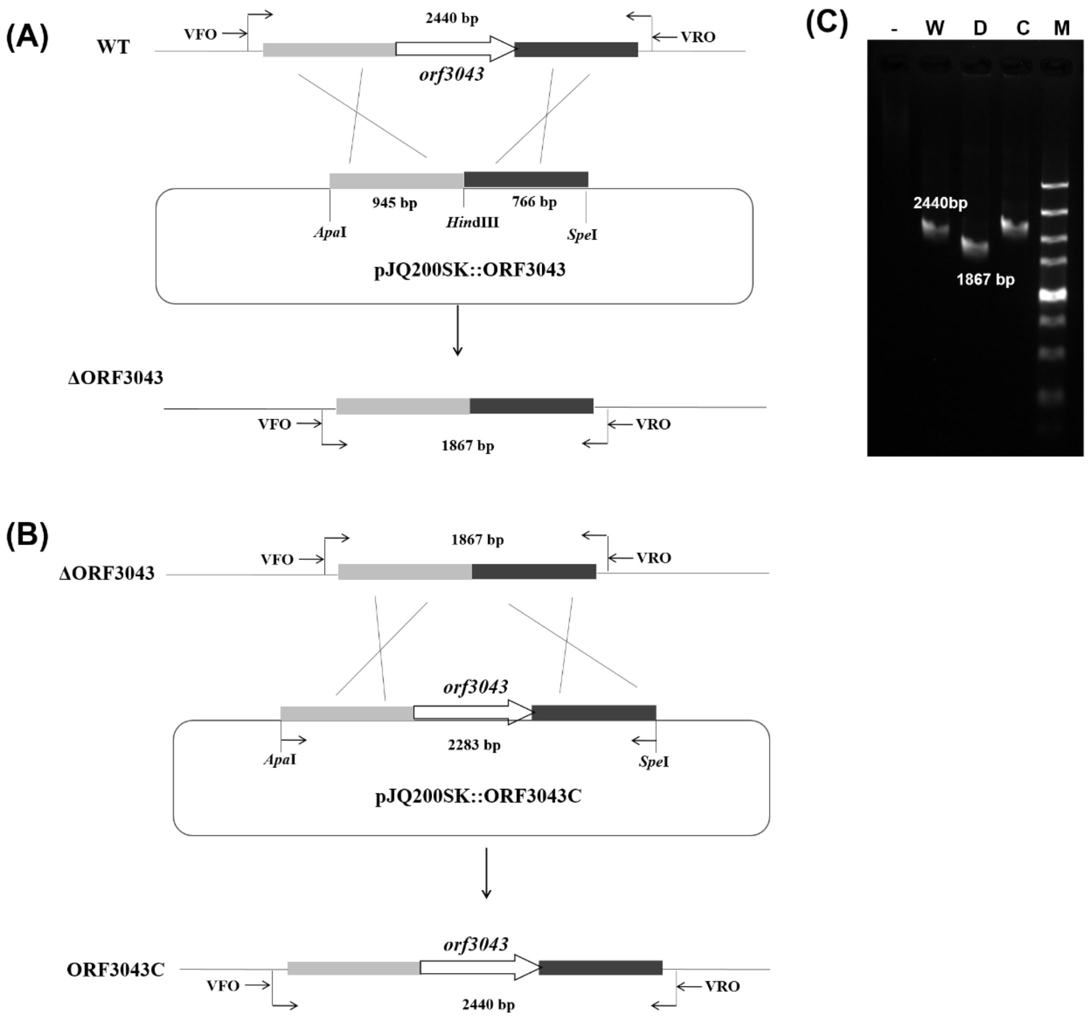

Figure S18. Generation of the deletion mutant ΔORF3043 of the TetR transcriptional regulator gene *orf3043* and its complementary strain ORF3043C. (A) Schematic representation of deleting *orf3043* in strain 3655 (WT). (B) Schematic representation of complementing *orf3043* in strain ΔORF3043. (C) PCR verification of ΔORF3043 and ORF3043C. -: negative control, H_2O used as the template; W: positive control (2440 bp expected), PCR product using VFO/VRO as the primers and genomic DNA of WT as templates; D: PCR product (1867 bp expected) using VFO/VRO as the primers and genomic DNA of ΔORF3043 as templates; C: PCR product (2440 bp expected) using VFO/VRO as the primers and genomic DNA of the complementary strain as templates; M: DNA marker.

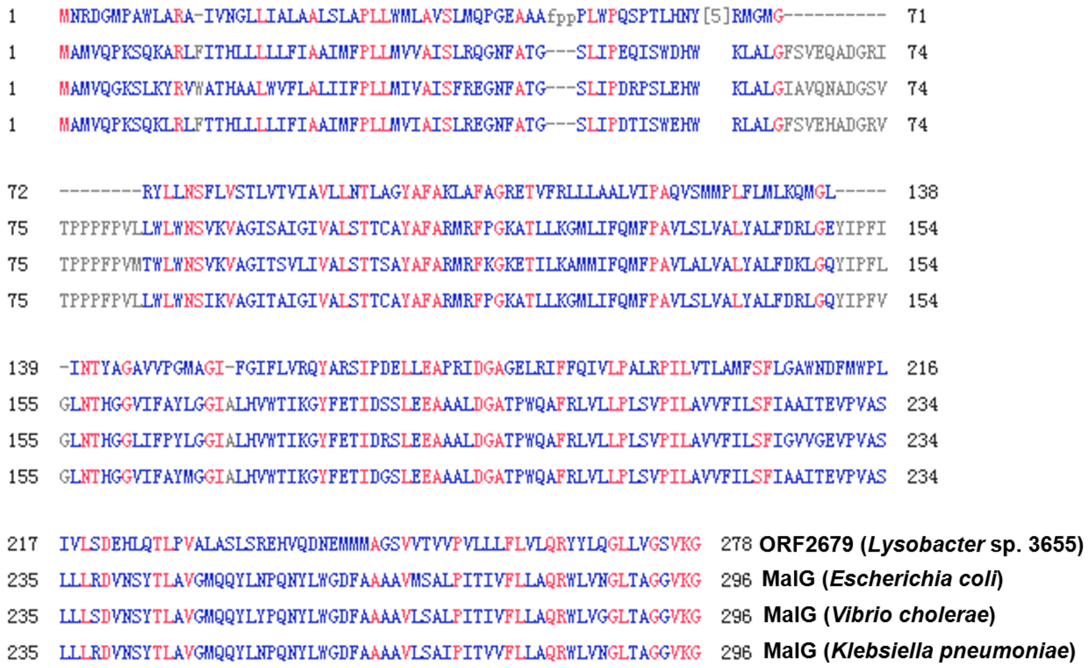

Figure S19. Alignment of the amino acid sequence of ORF2679 (PP824361) in strain 3655 with MalG proteins from *Escherichia coli*, *Vibrio cholerae*, and *Klebsiella pneumoniae*. MalG is one of the proteins in the maltose uptake system that is encoded by *malEFGK* genes. The red color indicates the identical residues, and the blue color indicates the highly conserved ones.

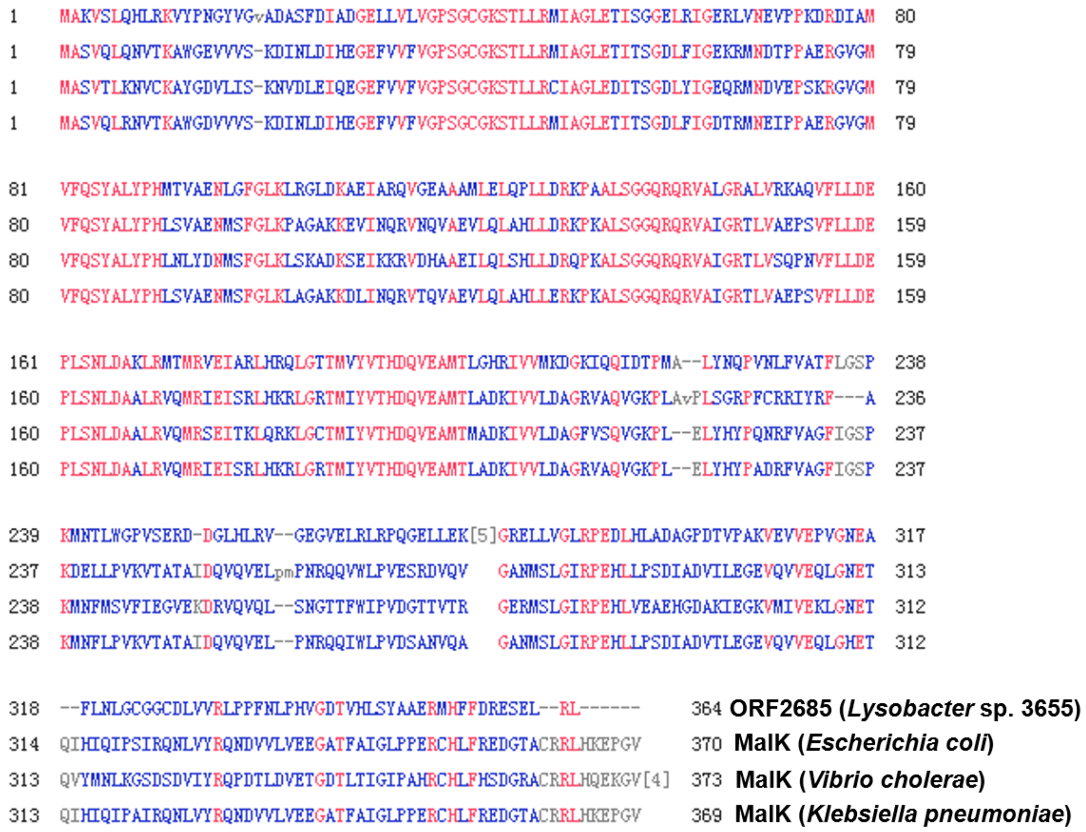

Figure S20. Alignment of the amino acid sequence of ORF2685 (PP824362) in strain 3655 with MalK proteins from *Escherichia coli*, *Vibrio cholerae*, and *Klebsiella pneumoniae*. MalK is one of the proteins in the maltose uptake system that is encoded by *malEFGK* genes. The red color indicates the identical residues, and the blue color indicates the highly conserved ones.

Table S1. Bacterial strains and plasmids used in this study

Bacterial strains/plasmids	Relevant characteristics[a]	Source/references
***Lysobacter* sp. 3655**		
3655	Wild-type, Km^r	DSM
ΔORF2903	The deletion mutant of *orf2903* in 3655	This study
ΔORF2904	The deletion mutant of *orf2904* in 3655	This study
ΔORF2905	The deletion mutant of *orf2905* in 3655	This study
ΔORF2906	The deletion mutant of *orf2906* in 3655	This study
ΔORF2677	The deletion mutant of *orf2677* in 3655	This study
ΔORF2678	The deletion mutant of *orf2678* in 3655	This study
ΔORF2742	The deletion mutant of *orf2742* in 3655	This study
ΔORF644	The deletion mutant of *orf644* in 3655	This study
ΔORF4279	The deletion mutant of *orf4279* in 3655	This study
ΔORF4990	The deletion mutant of *orf4990* in 3655	This study
ΔORF4991	The deletion mutant of *orf4991* in 3655	This study
ΔORF4992	The deletion mutant of *orf4992* in 3655	This study
ΔORF4993	The deletion mutant of *orf4993* in 3655	This study
ΔORF3043	The deletion mutant of *orf3043* in 3655	This study
ORF3043C	The complementary strain of ΔORF3043	This study
**Other bacteria**		
L. enzymogenes OH11	Wild-type, Km^r	Qian et al., 2009
Escherichia coli Trans 5α	Host strain for molecular cloning	Transgen
E. coli S17-1	Strain for conjugation with *Lysobacter*	Laboratory collection
**Plasmids**		
pJQ200SK	Cloning vector, Gm^r	Quandt and Hynes, 1993
pJQ200SK::ORF2903	Plasmid for the deletion of *orf2903*, Gm^r	This study
pJQ200SK::ORF2904	Plasmid for the deletion of *orf2904*, Gm^r	This study
pJQ200SK::ORF2905	Plasmid for the deletion of *orf2905*, Gm^r	This study
pJQ200SK::ORF2906	Plasmid for the deletion of *orf2906*, Gm^r	This study
pJQ200SK::ORF2677	Plasmid for the deletion of *orf2677*, Gm^r	This study
pJQ200SK::ORF2678	Plasmid for the deletion of *orf2678*, Gm^r	This study
pJQ200SK::ORF2742	Plasmid for the deletion of *orf2742*, Gm^r	This study
pJQ200SK::ORF644	Plasmid for the deletion of *orf644*, Gm^r	This study
pJQ200SK::ORF4279	Plasmid for the deletion of *orf4279*, Gm^r	This study
pJQ200SK::ORF4990	Plasmid for the deletion of *orf4990*, Gm^r	This study
pJQ200SK::ORF4991	Plasmid for the deletion of *orf4991*, Gm^r	This study
pJQ200SK::ORF4992	Plasmid for the deletion of *orf4992*, Gm^r	This study
pJQ200SK::ORF4993	Plasmid for the deletion of *orf4993*, Gm^r	This study
pJQ200SK::ORF3043	Plasmid for the deletion of *orf3043*, Gm^r	This study
pJQ200SK::ORF3043C	Plasmid for the complementary of *orf3043*, Gm^r	This study

[a]Km^r, kanamycin resistant; Gm^r, gentamicin resistant

Table S2. Primers used in this study

Primer	Sequence (5'-3')
ORF2903UF	CCGCTCGAGGCACTGGCAGACGAACTTG (*Xho*I)
ORF2903UR	CCCAAGCTTCTGGAGGAGAACAGCGAATC (*Hin*dIII)
ORF2903DF	CCCAAGCTTCCGCTGTGCCTTGATTGAG (*Hin*dIII)
ORF2903DR	GGACTAGTGGCTGCTGAAGTCGTAACG (*Spe*I)
ORF2903VFI	AACTGCTCGGGCAACTGCT
ORF2903VRI	TCGGTCGGATAACCGCACA
ORF2904UF	CCGCTCGAGTGTTCTCCTCCAGCCAGAC (*Xho*I)
ORF2904UR	CCCAAGCTTGATAGCGTGCGGCGAATT (*Hin*dIII)
ORF2904DF	CCCAAGCTTCCGCTTCCTCTTTCGTCC (*Hin*dIII)
ORF2904DR	GGACTAGTCAATCGCTGCGTTATGCC (*Spe*I)
ORF2904VFI	ATGGCGACACCGTCTACCT
ORF2904VRI	GAGCAGATAGCCTTCCACTTCT
ORF2905UF	CCGCTCGAGCCCGTGCTTGTGTTATCG (*Xho*I)
ORF2905UR	CCCAAGCTTGCGATCTTGGGAATGGTC (*Hin*dIII)
ORF2905DF	CCCAAGCTTCATCGGCATGGTCGAACTG (*Hin*dIII)
ORF2905DR	GGACTAGTACGGTGAGGTCCAGGTCTT (*Spe*I)
ORF2905VFI	GGTGCTGTTGATCCACGAT
ORF2905VRI	CGCCATCCGATGTTCTTCT
ORF2906UF	CCGCTCGAGCCGAAGAAGTGGAAGGCTATC (*Xho*I)
ORF2906UR	CCCAAGCTTATCGCAGGCCATCAAGGT (*Hin*dIII)
ORF2906DF	CCCAAGCTTCAACGCTTGGACGAACTC (*Hin*dIII)
ORF2906DR	GGACTAGTGCGAAACGATGTTGCAAC (*Spe*I)
ORF2906VFI	TTCGCCTATCTGCTGCTGAT
ORF2906VRI	ACGGTGAGGTCCAGGTCTT
ORF2677UF	CGGGGCCCGCTGATGATGGGCGTGCT (*Apa*I)
ORF2677UR	CCCAAGCTTCCAGCAGGACCGTCAGCA (*Hin*dIII)
ORF2677DF	CCCAAGCTTCCGACCGCATCTTGGAGAA (*Hin*dIII)
ORF2677DR	GGACTAGTCGAGACCAGGAAGCTGTTGA (*Spe*I)
ORF2677VFI	GGTGCCGTGGTATGTGGAA
ORF2677VRI	ACCGTGGAACTGCATCTGG
ORF2678UF	CGGGGCCCTGTCTGAGTTCGCCTTGCT (*Apa*I)
ORF2678UR	CCCAAGCTTCGAAGAACACGCCGATCAC (*Hin*dIII)
ORF2678DF	CCCAAGCTTTCCTGCTGTTCGTGCTGAT (*Hin*dIII)
ORF2678DR	GGACTAGTGAATGACGGTGTGACGGTAC (*Spe*I)
ORF2678VFI	GCGTGTTCAAGCCGTTCT
ORF2678VRI	GAAGCCTTCCTCGTACATCAG
ORF2742UF	CGGGGCCCCGCTGATGGTCCAGTCGC (*Apa*I)
ORF2742UR	CCCAAGCTTACGCTTTCGTTTCGCCCT (*Hin*dIII)
ORF2742DF	CCCAAGCTTGGATCGGGTGCTGTTCGTA (*Hin*dIII)
ORF2742DR	GGACTAGTTCTACCAGAACGCCTACTTGT (*Spe*I)
ORF2742VFI	TGCGGTTGGTATAGAAGTTGTT

ORF2742VRI	CGAATCGGTGGTGCTGGAA
ORF644UF	CCGCTCGAGGGGCAACTTCAACTCCAC (*Xho*I)
ORF644UR	CCCAAGCTTTTGCTGGTGGAGATCGAC (*Hin*dIII)
ORF644DF	CCCAAGCTTGTCGGCGTCGAGGTAATC (*Hin*dIII)
ORF644DR	GGACTAGTCGCCGTAGTTGAGGATGT (*Spe*I)
ORF644VFI	ACAGCAACAGCGGCATCT
ORF644VRI	GAACTACCTCGACGGCATCA
ORF4279UF	CCGCTCGAGGGCGTTGGAGACCAGGAT (*Xho*I)
ORF4279UR	CCCAAGCTTCGTGGCGTAGTCGGTGAG (*Hin*dIII)
ORF4279DF	CCCAAGCTTCTCAGCAGCGTCACCCTC (*Hin*dIII)
ORF4279DR	GGACTAGTGTCTTCCTCGCCGATACC (*Spe*I)
ORF4279VFO	CAGTTCCTTGGTGTGCTTCTC
ORF4279VRO	CTGCGGCTCGTTCTTCTTG
ORF4990UF	CCGCTCGAGGGCGGTTTCGAAGTCGTC (*Xho*I)
ORF4990UR	CCCAAGCTTCAGCGAGGTGAGGACAGC (*Hin*dIII)
ORF4990DF	CCCAAGCTTCAGTTGCTGGGCGTCTCC (*Hin*dIII)
ORF4990DR	GGACTAGTGGATGTGAGCGGCGTTTG (*Spe*I)
ORF4990VFI	TACCTCAGCCGCTACCTCGA
ORF4990VRI	GCGGTTGAGCAGGAAGATCC
ORF4991UF	CGGGGCCCGCAGCAGCGTCTTAGTTC (*Apa*I)
ORF4991UR	CCCAAGCTTGCTGAGTTCGGATTCGTC (*Hin*dIII)
ORF4991DF	CCCAAGCTTGTGTGGACGCTGGGCTCG (*Hin*dIII)
ORF4991DR	GGACTAGTCGGTCGGATTGCTGGGAA (*Spe*I)
ORF4991VFI	AACGACGGTCACCGCAAT
ORF4991VRI	TCTTCGCCATAACTGACCCATT
ORF4992UF	CCGCTCGAGCTTCCTGCTCAACCGCTTC (*Xho*I)
ORF4992UR	CCCAAGCTTACATGCAACGAACTCCTCAAG (*Hin*dIII)
ORF4992DF	CCCAAGCTTACGGCGATTACGACCTCTATC (*Hin*dIII)
ORF4992DR	GGACTAGTGGCGAATGAATACAGGCACAG (*Spe*I)
ORF4992VFO	CGGTCGTCCTGTAGTTCGTA
ORF4992VRO	GCTTGTTGCTGCCGTTGTA
ORF4992VFI	GCCAACAACCGCAACTACAT
ORF4992VRI	TGAGCAGCCACGATTCCAT
ORF4993UF	CCGCTCGAGCGCTCGGACGACGAATAC (*Xho*I)
ORF4993UR	CCCAAGCTTGGCGGAGAGAGTGAGGCT (*Hin*dIII)
ORF4993DF	CCCAAGCTTGGCACCAAGAATCCCTGA (*Hin*dIII)
ORF4993DR	GGACTAGTGATGCGGTTGTGCTTGAA (*Spe*I)
ORF4993VFI	ACCAGAGCGACAACACCTAC
ORF4993VRI	TGCCGATGTTCACCGTCAC
ORF3043UF	CGGGGCCCCCAGTTGGCGTTGTAGGC (*Apa*I)
ORF3043UR	CCCAAGCTTCATGGCGGCAAGGTTACA (*Hin*dIII)
ORF3043DF	CCCAAGCTTACGGCGACGGATATGTTC (*Hin*dIII)
ORF3043DR	GGACTAGTCACCTTCCCAGGAGCAAA (*Spe*I)
ORF3043VFO	GAAGGTGCCGTTGTGGAT

ORF3043VRO	GAGGTCATAGCGTTTGCG
ORF2903-real-F	AACTGGCGTTGGTGTCGA
ORF2903-real-R	GTCTGGCTGGAGGAGAACA
ORF2905-real-F	CTGATCGTCTGCGGCATCTA
ORF2905-real-R	CGCCATCCGATGTTCTTCTG
ORF644-real-F	AGATGCCGCTGTTGCTGTC
ORF644-real-R	CCGATCAGGCTGTCGAAGTT
ORF3043-real-F	GCACCGTCTACAACCACTTC
ORF3043-real-R	TGGACCAGGTCGAGCAACT
ORF4279-real-F	ATCGTCGAGGCGATGGAA
ORF4279-real-R	CGGCTGTTTCGGTTGGAA
ORF4990-real-F	CGATGTGGCTGGAGGAAGA
ORF4990-real-R	AAGCGGTTGAGCAGGAAGA
ORF4992-real-F	CCTACGCCTACCGCAAGAA
ORF4992-real-R	TGAGCAGCCACGATTCCAT
ORF4993-real-F	CGTGTTCCACTTCGCCAAG
ORF4993-real-R	TTGTTGCTGCCGTTGTACTG
3655-16S-real-F	TCCACGCCCTAAACGATG
3655-16S-real-R	TTGCAGCCCTCTGTCCCT

Table S3. Deduced function of genes in Cluster 7 (PP824363) of *Lysobacter* sp. 3655

ORF	Size (aa)	Annotation	%identity/ similarity	Accession number
orf2903	421	isochorismate synthase	97.62/100	WP_211664563.1
orf2904	549	(2,3-dihydroxybenzoyl) adenylate synthase	97.63/100	WP_096378120.1
orf2905	292	isochorismatase family protein	98.97/100	WP_139175066.1
orf2906	437	condensation domain-containing protein	98.17/100	WP_074870215.1
orf2907	280	siderophore-interacting protein	96.07/100	WP_074870344.1
orf2908	252	2,3-dihydro-2,3-dihydroxybenzoate dehydrogenase	98.41/100	WP_096378132.1

Table S4. The upregulated iron-related genes from transcriptomics data

Gene	Gene id	MeanTPM[β] (S)	MeanTPM (DS)	log2FoldChange	qValue[γ]
orf4279	TR3851	95.98027	5.48053	4.13035	8.68E-09
orf4990	TR2489	54.13147	7.76294	2.801792	5.57E-194
orf4991	TR2489	54.13147	7.76294	2.801792	5.57E-194
orf4992	TR0501	319.8193	11.69751	4.772984	3.63E-123
orf4993	TR3399	12368.05	183.999	6.070776	5.31E-194
orf4994	TR3405	36.24963	4.36527	3.053824	8.67E-107

[β]TPM, Transcripts Per Million; [γ]qValue, Corrected *P* Value

Table S5. Deduced function of upregulated iron-related genes

ORF	Size (aa)	Annotation	%identity/ similarity	Accession number
orf4279	153	SUF system Fe-S cluster assembly regulator	100/100	WP_074864001.1
orf4990	167	RpoE family RNA polymerase sigma factor	93.98/99	WP_207523027.1
orf4991	351	FecR domain-containing protein	96.58/99	WP_074870855.1
orf4992	1017	TonB-dependent transporter	98.92/99	WP_096381001.1
orf4993	247	Slam-dependent surface lipoprotein, transferrin-binding B	99.59/99	WP_074870853.1
orf4994	494	surface lipoprotein assembly modulator	97.36/99	WP_083382851.1

1. Qian, G. L., Hu, B. S., Jiang, Y. H., and Liu, F. Q. (2009) Identification and characterization of *Lysobacter enzymogenes* as a biological control agent against some fungal pathogens. *Agric. Sci. China 8,*68–75.

2. Quandt, J., and Hynes, M. F. (1993) Versatile suicide vectors which allow direct selection for gene replacement in gram-negative bacteria. *Gene 127,* 15–21.
